# Comparison of meglumine antimoniate versus miltefosine in the treatment of new world cutaneous leishmaniasis: a systematic review and meta-analysis^☆^

**DOI:** 10.1016/j.abd.2025.501253

**Published:** 2026-01-08

**Authors:** Ana Carolina Putini Vieira, Fernanda Cronemberger Lins, Arianne Costa Baquião

**Affiliations:** Department of Medicine, Universidade Santo Amaro, São Paulo, SP, Brazil

**Keywords:** Drug therapy, Leishmaniasis, Cutaneous, Meglumine antimoniate

## Abstract

**Background:**

Cutaneous Leishmaniasis (CL) affects up to 1.2 million people annually, mainly in resource-limited regions. Meglumine antimoniate, the standard treatment, is limited by systemic toxicity, injectable administration, and increasing resistance. Miltefosine, an oral alternative, offers practical advantages, although comparative efficacy and safety data remain inconsistent.

**Objective:**

To compare the efficacy and safety of miltefosine versus meglumine antimoniate for New World CL.

**Methods:**

The authors systematically searched PubMed, Embase, Scopus, and the Cochrane Library for randomized controlled trials directly comparing miltefosine and meglumine antimoniate. Risk Ratios (RRs) with 95% Confidence Intervals (95% CIs) were calculated using random-effects models. Heterogeneity was assessed with the I² statistic. Risk of bias was evaluated using the Cochrane RoB-2 tool. Certainty of evidence was assessed using the Grading of Recommendations, Assessment, Development, and Evaluations (GRADE) approach.

**Results:**

Eight trials involving 898 patients (502 treated with miltefosine, 396 with meglumine antimoniate) were included. Miltefosine showed significantly higher cure rates at two months (RR = 0.83; 95% CI: 0.71–0.98; *I*^2^ = 0%). Differences at six months were not statistically significant. Gastrointestinal side effects were more frequent with miltefosine, whereas hepatic enzyme elevations, arthralgia (RR = 10.08; 95% CI: 2.36–43.12), and fever (RR = 2.98; 95% CI: 1.53–5.80) were more common with meglumine antimoniate.

**Study Limitations:**

High heterogeneity, short follow-up, small sample sizes, and interstudy variability may limit precision.

**Conclusion:**

Miltefosine shows superior early response and a safer systemic profile. However, the certainty of evidence, as assessed by GRADE, ranged from very low to high across outcomes, and long-term data remain limited, highlighting the need for further high-quality studies with extended follow-up.

## Introduction

Cutaneous leishmaniasis (CL) is a neglected tropical disease (NTD) that primarily affects impoverished populations in tropical and subtropical regions worldwide. It is broadly categorized into two main forms: Old World CL, occurring in parts of Africa, Europe, and Asia, and New World CL. These forms differ significantly in geographic distribution, *Leishmania* species, clinical manifestations, and treatment response.^1^

New World CL, also referred to as American CL (ACL) or American Tegumentary Leishmaniasis (ATL), is especially prevalent in tropical and subtropical areas of the Americas. The infection is transmitted by the bite of *Lutzomyia* sandflies and typically leads to persistent skin ulcers that may result in significant scarring. In more severe cases, the disease can extend to the mucous membranes of the nose, mouth, and throat.^2^, ^3^

Multiple *Leishmania* species are responsible for ACL, with *Leishmania (Viannia) braziliensis*, *Leishmania (Leishmania) amazonensis*, and *Leishmania (Viannia) guyanensis* being the most commonly identified in Brazil.^2^ These parasites, classified within the *Leishmania* and *Viannia* subgenera, show notable differences in their capacity to cause severe disease and in their responsiveness to treatment options.^3^

This biological and clinical diversity contributes to the difficulty of controlling ACL, especially in endemic countries such as Brazil, Colombia, and Peru.^4^, ^5^ This complexity is reflected in the substantial and persistent burden of disease. Although it is estimated that between 600,000 and 1 million new CL cases occur globally each year, only about 200,000 are officially reported to the World Health Organization (WHO).^6^

In 2023 alone, 272,098 new CL cases were reported, with 94% originating from the Eastern Mediterranean Region and the Americas. Brazil, alongside Afghanistan, Algeria, Colombia, Iran, Iraq, Pakistan, Peru, Sri Lanka, Syria, and Yemen, accounted for over 90% of all globally reported cases. Notably, case numbers in the Americas have rebounded following declines during the COVID-19 pandemic, reflecting both renewed transmission and improvements in case detection. Despite this, underreporting remains a persistent issue due to limited surveillance infrastructure, barriers to healthcare access, and variations in national reporting systems.^7^

Given the substantial disease burden and the considerable variability in clinical presentation and *Leishmania* species across endemic regions, the treatment of CL remains particularly challenging.^8^ Pentavalent antimonials (SbV) have been the primary treatment for leishmaniasis since 1945, with meglumine antimoniate being the most commonly used. Although the mechanism of action of SbV is not fully understood, it is believed that its antileishmanial activity is due to the stimulation of the host's macrophages. However, the use of SbV is associated with serious adverse effects, including hepatotoxicity, cardiotoxicity, and nephrotoxicity.^9^, ^10^ Since the 1980s, resistance to meglumine antimoniate has increased, largely due to inappropriate use.^11^ Furthermore, its parenteral administration presents additional challenges, particularly in remote and resource-limited areas where adherence to treatment regimens can be difficult.^10^

Miltefosine, an oral medication, is an alternative in cases of antimonial resistance. Its mechanism of action involves interfering with the lipids in the membrane of the *Leishmania* parasite and its mitochondrial function. Studies suggest miltefosine may be better tolerated compared to other treatments. Although there are also reports of side effects such as vomiting, diarrhea, and, to a lesser extent, hepatotoxicity and nephrotoxicity.^11^ A major limitation of miltefosine is its teratogenic potential, compounded by its prolonged persistence in the body for up to four months after treatment.^12^

Notwithstanding the availability of these therapies, relapse remains common. Parasitic resistance and incomplete eradication of *Leishmania*, particularly its persistence in scar tissue, may contribute to disease recurrence. Almeida-Santos et al., in a systematic review, reported relapse rates of 52% after a single drug, with 45% of patients treated with Glucantime (meglumine antimoniate), alone or in combination, experiencing treatment failure, most often defined between 6–12 months after treatment. These findings highlight the ongoing difficulty in achieving sustained parasitological cure in CL.^13^

Although the efficacy of miltefosine has been demonstrated in various studies, the comparison between miltefosine and meglumine antimoniate remains limited and inconsistent, as different studies report varying results regarding the efficacy of the two drugs.^14^ Given the toxicity of antimonial drugs and the difficulty of their use in remote areas, miltefosine presents an important alternative for the treatment of CL, especially in low-income populations, due to its ease of administration.^10^

While this comparison is clinically relevant, no comprehensive synthesis has yet resolved the conflicting evidence regarding the relative efficacy and safety of meglumine antimoniate and miltefosine across diverse settings. Several prior meta-analyses, published between 2013 and 2021, have examined aspects of this question. However, most combined studies from both Old World and New World cutaneous leishmaniasis, despite the significant differences in species distribution, clinical presentation, and treatment response between the two regions.^14^, ^15^, ^16^, ^17^ Consequently, their findings lacked geographic specificity and provided limited guidance for treatment decisions in the Americas.

The present analysis will specifically address this gap by focusing solely on New World CL, evaluating cure rates from early time points through long-term follow-up and systematically assessing treatment failure rates, an outcome often underreported in previous studies. Therefore, this systematic review and meta-analysis aims to compare the efficacy and toxicity of meglumine antimoniate and miltefosine for the treatment of New World cutaneous leishmaniasis.

## Methods

This systematic review was conducted in accordance with the protocols established by the Cochrane Collaboration and adhered to the guidelines outlined in the Preferred Reporting Items for Systematic Reviews and Meta-Analyses (PRISMA).^18^, ^19^ The study protocol was preregistered in the International Prospective Register of Systematic Reviews (PROSPERO) under the identification number CRD420251044262.

### Eligibility criteria and study selection

The eligibility criteria were structured according to the PICOS framework: Population: patients with New World CL; Intervention: miltefosine; Comparison: meglumine antimoniate; Outcomes: cure rates at various follow-up points, treatment failure, and adverse events; Study type: Randomized Controlled Trials (RCTs).

Two reviewers (A.C.P.V. and F.C.L.) independently screened the articles for inclusion, resolving any discrepancies through consensus. Inclusion in this systematic review was restricted to studies that met all the following eligibility criteria: (1) RCTs; (2) In vivo studies; (3) Human studies; and (4) Direct comparisons of miltefosine and meglumine antimoniate for the treatment of New World CL. Exclusion criteria included: (1) Review articles, case reports, case series, observational studies and non-randomized clinical trials; (2) Studies that did not explicitly specify meglumine antimoniate as the pentavalent antimonial used; (3) Unpublished or incomplete clinical trials; (4) In vitro studies; (5) Studies that did not directly compare miltefosine to meglumine antimoniate; (6) Studies on old world cutaneous leishmaniasis; and (7) Duplicate publications.

### Search strategy and data extraction

The authors systematically searched PubMed, Embase, Scopus and Cochrane Library databases, from inception to November 1, 2024. The search strategy used was (“Meglumine Antimoniate” OR “Glucantime” OR “N-Methylglucamine Antimonate”) AND (“Miltefosine” OR “hexadecylphosphocholine” OR “Impavido” OR “Miltex”) AND (“Cutaneous Leishmaniasis” OR “American cutaneous leishmaniasis” OR “tegumentary leishmaniasis” OR CL OR ACL OR “skin” OR “dermal leishmaniasis”).

Following the removal of duplicates, the titles and abstracts of the remaining studies were screened in Rayyan. The studies’ titles and abstracts were reviewed based on eligibility criteria. Subsequently, selected papers underwent a thorough examination by full-text reading. These screening processes were carried out independently by two reviewers (A.C.P.V. and F.C.L.) to minimize bias. Disagreements were addressed through discussion and consensus by the two reviewers.

Two authors (A.C.P.V. and F.C.L.) independently extracted data to obtain the following information from each study: (1) Study characteristics: name of authors, year of publication, country of origin, parasite species, inclusion criteria, number of patients, age of patients, follow-up, interventions; (2) Outcomes: cure rates at 1-, 2-, 3-, 4-, 6-, and 12-months, cure failure at 6-months, cure rates at 2-, 3- and 6-months in *L.braziliensis* infections, vomiting, nausea, abdominal pain, and diarrhea, Alanine Aminotransferase (ALT), Aspartate Aminotransferase (AST), arthralgia, fever, and headache. Other adverse effects, such as cardiac and renal changes, were not evaluated due to insufficient data across the included studies to allow for statistical analysis. Discrepancies in data extraction were resolved by consensus.

### Quality assessment

Two authors (A.C.P.V. and F.C.L.) assessed the quality of the included studies. As suggested by Cochrane, risk of bias was assessed using the Cochrane risk-of-bias tool for randomized trials (RoB-2).^20^ Studies included in this meta-analysis were classified as having a low risk of bias and some concerns for risk of bias. In addition, the overall quality of evidence was assessed following the Grading of Recommendations, Assessment, Development, and Evaluations (GRADE) guidelines.^21^ Studies were categorized as having very low, low, moderate, or high-quality evidence on the basis of considerations including risk of bias, inconsistency of results, imprecision, publication bias, and magnitude of treatment effects.

### Statistical analysis

The statistical analysis was performed using *R* statistical software version 4.5.0 (R Foundation for Statistical Computing). The following packages were used: “metaprop”, “metafor, “dmetar”, “ggplot2”, and “meta”. The outcomes were evaluated using proportions with 95% Confidence Intervals (95% CI). According to Cochrane's recommendations, a random-effects model was used for all outcomes, accounting for variability between studies. The Cochrane *Q* test and I2 statistics were performed to quantify heterogeneity. Endpoints were considered to have low heterogeneity if *I*^2^ < 25%. To minimize heterogeneity and detect outliers, sensitivity analysis using “leave-one-out” was conducted. Additionally, Baujat plots were generated for outcomes presenting moderate to high heterogeneity (*I*^2^ > 25%) to identify studies contributing most to heterogeneity and influence.

## Results

### Selection of studies

**Fig. 1:**
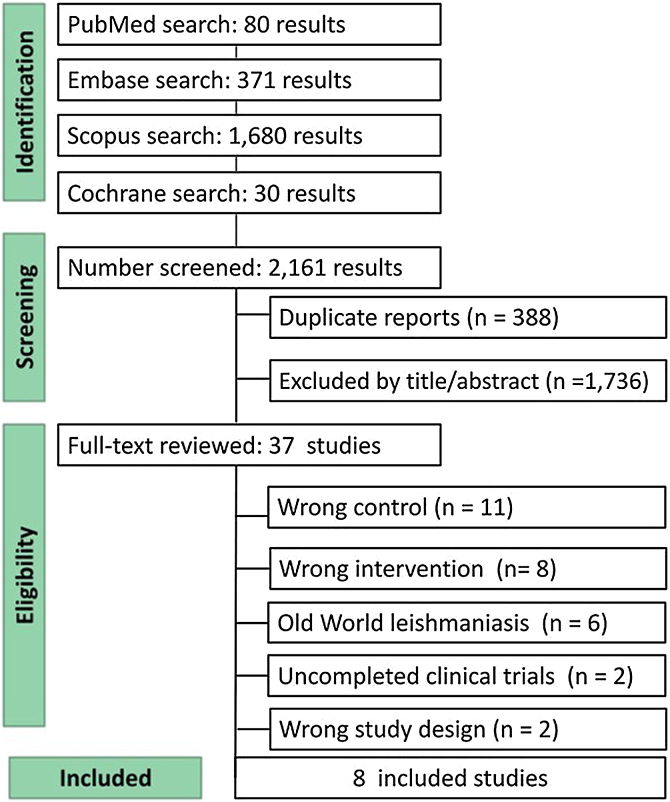
PRISMA search flow diagram.

As depicted in Fig. 1, this search identified 2.161 results: 80 from PubMed, 371 from Embase, 1,680 from Scopus, and 30 from Cochrane Library. Of these, 388 were identified as duplicates, and 1,736 were excluded based on their title and/or abstract for not meeting the inclusion criteria. Subsequently, 37 studies underwent full-text review, of which 8 RCTs met the eligibility criteria and were included in this systematic review. A total of 898 patients were included, with 396 receiving meglumine antimoniate and 502 receiving miltefosine.^22^, ^23^, ^24^, ^25^, ^26^, ^27^, ^28^, ^29^

### Pooled analysis of all studies

The present analysis included eight RCTs evaluating the efficacy of meglumine antimoniate and miltefosine for the treatment of CL. Participants were aged 0 to 65 years, and all had confirmed diagnoses of CL. The most frequently identified parasite species was *L. braziliensis*. Follow-up periods ranged from 1 to 12 months, with most studies reporting outcomes at 3 and 6 months post-treatment.^22^, ^23^, ^24^, ^25^, ^26^, ^27^, ^28^, ^29^ Study characteristics are detailed in Table 1.^22^, ^23^, ^24^, ^25^, ^26^, ^27^, ^28^, ^29^

**Table 1:** Baseline characteristics of the included studies.

Study, year	Study Design	Parasite Species	Inclusion Criteria	Nº of Patients MF/MA	Age Range (years)	Follow - Up	Interventions
Chrusciak Talhari et al. 2011^22^	RCT	*L. guyanensis*	1‒5 lesions, 1 ulcerated, < 3-months, Leishmania amastigotes in biopsy, no prior treatment	56 / 28	2 ‒ 65	6 and 12 mo	MA: IV 20 mg (13‒65 yrs) or 15 mg (2‒12 yrs) for 20 days (max 3 ampoules/day).
		*L. braziliensis*					
		*L. lainsoni*					MF: Oral 2.5 mg/kg daily for 28 days
							
Machado et al. 2010^23^	RCT	*L. braziliensis*	Typical ulcer, positive Montenegro test, in endemic area; age 2‒65; up to 5 ulcers, 2 regions; 10‒50 mm; < 90 days since first ulcer.	60 / 30	4 ‒ 65	2 w, 1, 2, 4 and 6 mo	MA: IV 20 mg SbV/kg/day for 20 days (max 3 ampoules or 1,215 mg SbV/day).
							MF: Oral 2.5 mg/kg BW (max150 mg) daily for 28 days
							
Machado et al. 2021^24^	RCT	*L. braziliensis*	Age 18–65, 1–3 ulcers (10–50 mm), < 90 days since onset.	47 / 45	4 ‒ 65	2 and 6 mo	MA: IV 20 mg SbV/kg/day for 20 days.
							MF: Oral 2.5 mg/kg BW (max150 mg) daily for 28 days + Placebo
							
Mendes et al. 2020^25^	RCT	*L. guyanensis*	Age 18–65 years, 1–5 ulcers (10–50 mm), illness duration 30–90 days, no prior treatment.	50 / 50	27 ‒ 50	2, 3 and 6 mo	MA: IV 20 mg SbV/kg/day for 20 days.
		*L. braziliensis*					
		*L. naifi*					MF: Oral 2.5 mg/kg BW (max150 mg) daily for 28 days + Placebo
							
Rubiano et al. 2012^26^	RCT	*L. panamensis/ guyanensis*	Parasitologically confirmed CL	58 / 58	2 ‒ 12	6 mo	MA: (81 mg Sb/mL) 20 mg Sb/kg/ day IM for 20 days.
							MF: 10 mg/capsule, 1.5–2.5 mg/kg/ day orally for 28 days, divided into 2‒3 doses
							
Soto et al. 2007^27^	RCT	*L. braziliensis*	NA	45 / 26	NA	2,4 and 6 mo	MA: IM for 28 days
							MF: Oral for 28 days
							
Soto et al. 2008^28^	RCT	*L. braziliensis*	*Leishmania*-positive ulcer (Giemsa), 12+, no ML, no treatment in 6-months, no significant comorbidities	41 / 16	≥12	1, 3, 6 and 12 mo	MA: IM 20 mg/kg/d for 20 days.
							MF: Oral 2.5 mg/kg/d for 28 days
							
Vélez et al. 2010^29^	RCT	*L. (V.) panamensis / braziliensis*	Confirmed leishmaniasis; no treatment in 6-weeks; normal renal, hepatic, and hematological functions.	145 / 143	19 ‒ 38	6 mo	MA: IM 20 mg/kg/day for 20 days
							MF: 50 mg capsule was taken 3 times a day for 28 days

RCT, Randomized Controlled Trial; NA; Not Available; CL, Cutaneous Leishmaniasis; ML, Mucosal Leishmaniasis; MA, Meglumine Antimoniate; MF, Miltefosine; ± mean or median; IV, Intravenous; IM, Intramuscular.

### Efficacy outcomes over time

**Fig. 2:**
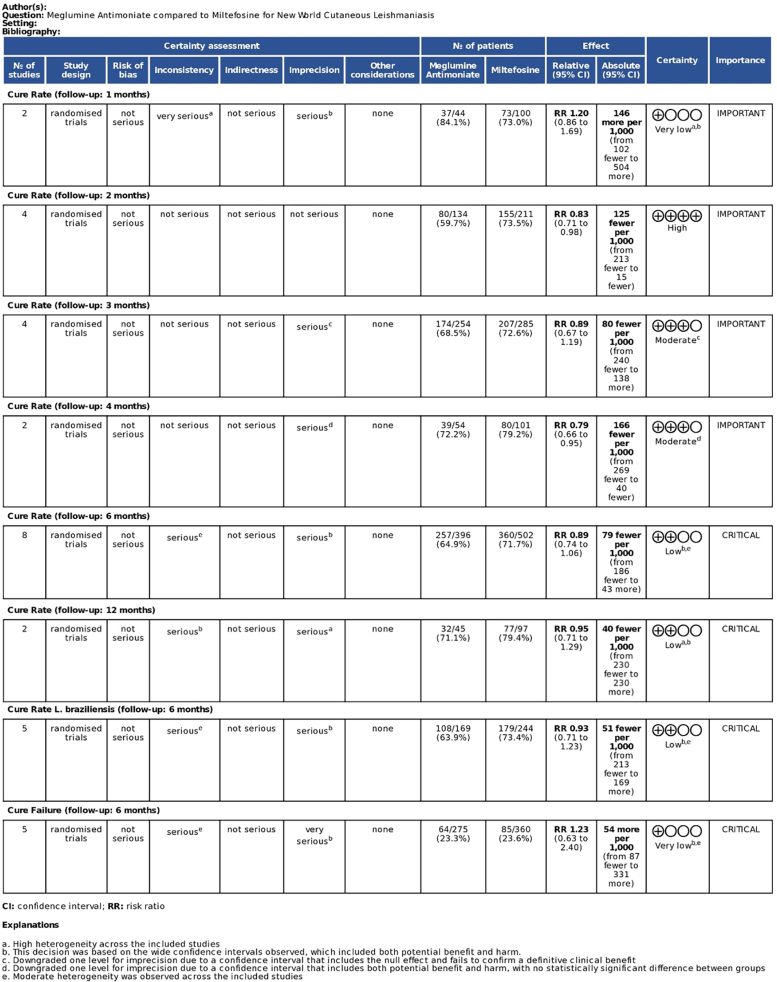
GRADE assessment ‒ efficacy outcomes.

Regarding efficacy outcomes, no significant difference was observed between the groups in cure rates at 1-month (RR = 1.20; 95% CI: 0.86, 1.69; p = 0.283; *I*^2^ = 77.3%; Supplemental Fig. S1). This meta-analysis included two studies with a total of 144 patients (73 treated with miltefosine and 44 treated with meglumine antimoniate).^22^, ^28^ While one study demonstrated a significant benefit with miltefosine (RR = 1.41; 95% CI: 1.17, 1.71),^28^ the overall effect was not statistically significant due to considerable heterogeneity (Chi^2^ = 4.40; p = 0.0359). Baujat plot analysis is available in the Supplemental Material (Supplemental Fig. S2). The certainty of evidence for this outcome was very low due to inconsistency and serious imprecision (Fig. 2).

**Fig. 3:**
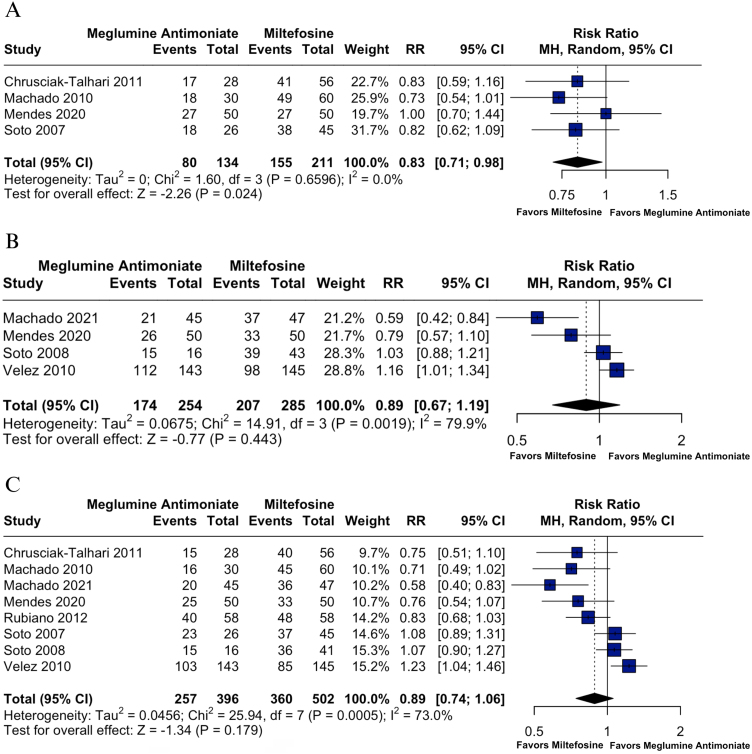
(A) Cure rates at 2-months post-treatment. (B) Cure rates at 3-months post-treatment. (C) Cure rates at 6-months post-treatment.

This trend shifted notably at 2-months, when miltefosine demonstrated a statistically significant higher cure rate compared to meglumine antimoniate (RR = 0.83; 95% CI: 0.71, 0.98; p = 0.024; I^2^ = 0%; Fig. 3A). Based on two studies encompassing 161 patients (105 treated with miltefosine and 56 with meglumine antimoniate),^23^, ^27^ this effect was consistent across trials, with no indication of heterogeneity (Chi^2^ = 1.60; p = 0.6596), reinforcing the robustness of the finding. Consistent results across studies and narrow confidence intervals supported a high certainty rating for this outcome (Fig. 2).

By 3-months, miltefosine continued to show a numerical advantage, although the difference did not reach statistical significance (RR = 0.89; 95% CI: 0.67, 1.19; p = 0.443; I^2^ = 79.9%; Fig. 3B). This analysis included four studies with a total of 345 patients, of whom 155 received miltefosine and 134 received meglumine antimoniate.^24^, ^25^, ^27^, ^29^ Leave-one-out sensitivity analyses demonstrated minimal changes in the pooled effect size. Heterogeneity decreased the most when Machado et al. 2021 was excluded (*I*^2^ = 57%).^26^ Baujat plot analysis indicated that Velez et al. 2010 contributed most to both heterogeneity and influence on the overall effect estimate, followed by Machado et al. 2021 (Supplemental Figs. S3‒S4).^24^, ^29^ The certainty of evidence was rated moderate due to imprecision and inconsistency (Fig. 2).

At 4-months, cure rates remained comparable between groups (RR = 0.95; 95% CI: 0.79, 1.13; p = 0.553; I^2^ = 12.5%; Supplemental Fig. S5). This analysis included two studies with a total of 155 patients, 101 treated with miltefosine and 54 with meglumine antimoniate. Both studies reported consistent results, and heterogeneity was low (Chi^2^ = 1.14; p = 0.2851).^22^, ^27^ Despite low heterogeneity, the confidence interval crossed the line of no effect, leading to a moderate certainty rating with one downgrade for imprecision (Fig. 2).

At 6-months, no significant difference was found in long-term cure rates, although it slightly favored miltefosine (RR = 0.89; 95% CI: 0.74, 1.06; p = 0.179; *I*^2^ = 73%; Fig. 3C). This analysis included eight studies comprising 898 patients, with 502 treated with miltefosine and 396 with meglumine antimoniate.^22^, ^23^, ^24^, ^25^, ^26^, ^27^, ^28^, ^29^ Leave-one-out analyses revealed moderate variability in effect estimates (RR range: 0.81 to 1.03). The exclusion of Velez et al. 2010 reduced heterogeneity, although the overall interpretation remained unchanged. Baujat plot analysis again identified Velez et al. 2010 as having the highest contribution to both heterogeneity and influence on the overall pooled effect, followed by Machado et al. 2021 (Supplemental Figs. S6‒S7).^24^, ^29^ Despite these influences, the overall result was stable. The certainty of evidence was low (Fig. 2).

At 12-months, no statistically significant difference in cure rates was observed between treatments (RR = 0.95; 95% CI: 0.71, 1.29; p = 0.755; *I*^2^ = 59.9%; Supplemental Fig. S8). This analysis was based on two studies, including 142 patients, with 97 treated with miltefosine and 45 with meglumine antimoniate.^22^, ^28^ Baujat plot analysis is available in the supplemental material (Supplemental Fig. S9). The certainty of evidence was low, due to the wide confidence intervals observed and the heterogeneity observed across the included studies (Fig. 2).

### Leishmania braziliensis

To further investigate potential subgroup differences, efficacy at 2-, 3-, and 6-months was also assessed specifically in patients infected with *L. braziliensis*.

**Fig. 4:**
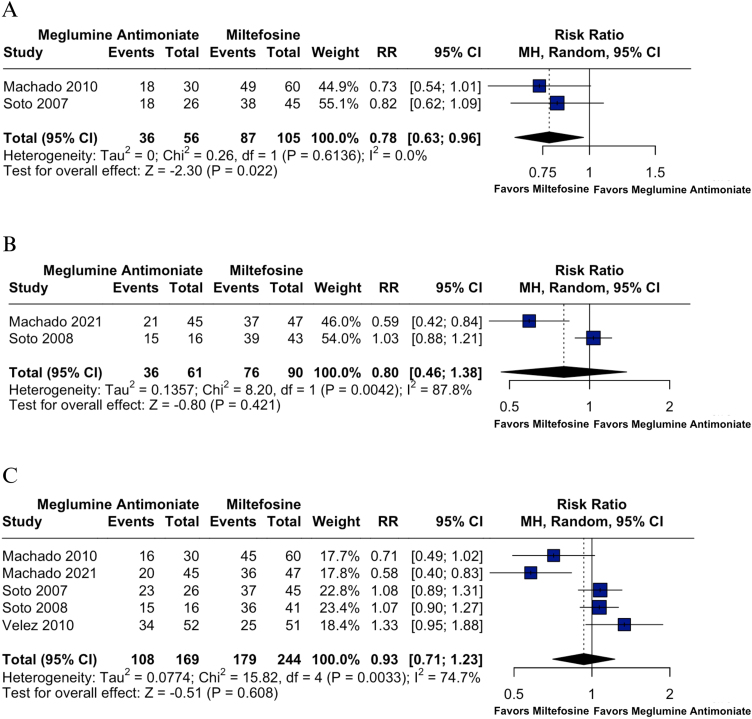
(A) Cure rates at 2-months post-treatment in *L. braziliensis* infections. (B) Cure rates at 3-months post-treatment in *L. braziliensis* infections. (C) Cure rates at 6-months post-treatment in *L. braziliensis* infections.

At 2-months, miltefosine demonstrated significantly higher cure rates compared to meglumine antimoniate (RR = 0.78; 95% CI: 0.63, 0.96; p = 0.022; *I*^2^ = 0%; Fig. 4A), with consistent findings across studies and no heterogeneity. This analysis was based on two studies encompassing 161 patients (105 treated with miltefosine and 56 with meglumine antimoniate).^23^, ^27^

**Fig. 5:**
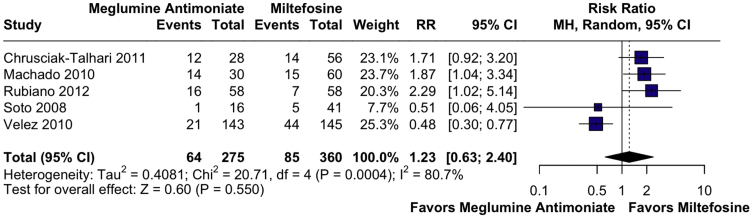
Cure failure at 6-months.

At 3-months, no statistically significant difference was found (RR = 0.80; 95% CI: 0.46, 1.38; p = 0.421; *I*^2^ = 87.8%; Fig. 4B). This analysis included two studies with a total of 151 patients, 90 treated with miltefosine and 61 with meglumine antimoniate.^24^, ^28^ Baujat analysis indicated inconsistency mainly due to Machado et al. 2021 (Supplemental Fig. S10).^24^

At 6-months, no statistically significant difference in cure rates was observed between miltefosine and meglumine antimoniate (RR = 0.93; 95% CI: 0.71, 1.23; p = 0.608; *I*^2^ = 74.7%; Fig. 4C). This analysis included five studies comprising 413 patients, with 244 treated with miltefosine and 169 with meglumine antimoniate.^23^, ^24^, ^27^, ^28^, ^29^ Leave-one-out sensitivity analysis showed that heterogeneity decreased most when Machado et al. 2021 was excluded (*I*^2^ = 52.7%), suggesting it contributed notably to heterogeneity. Consistently, the Baujat plot indicated that Machado et al. 2021 and Velez et al. 2010 were the main contributors to both heterogeneity and influence on the pooled result (Supplemental Figs. S11‒S12).^24^, ^29^

### Cure failure at 6-months

Cure failure at 6-months was also evaluated as a complementary efficacy outcome. In the pooled analysis, no statistically significant difference was found between miltefosine and meglumine antimoniate (RR = 1.23; 95% CI: 0.63, 2.40; p = 0.550; *I*^2^ = 80.7%; Fig. 5). This analysis included five studies encompassing 635 patients, with 360 treated with miltefosine and 275 with meglumine antimoniate.^22^, ^23^, ^26^, ^28^^,^^29^ Leave-one-out analysis confirmed the instability of the pooled estimate, with only the removal of Velez et al. 2010 markedly reducing heterogeneity (*I*^2^ = 0%) and shifting the result in favor of meglumine antimoniate (RR = 1.81; 95% CI: 1.25, 2.63), suggesting this study substantially influenced the overall effect. Baujat plot analysis again identified Velez et al. 2010 as having the highest contribution to both heterogeneity and influence on the overall pooled effect (Supplemental Figs. S13‒S14).^29^ The certainty of evidence was very low (Fig. 2).

### Safety outcomes

**Fig. 6:**
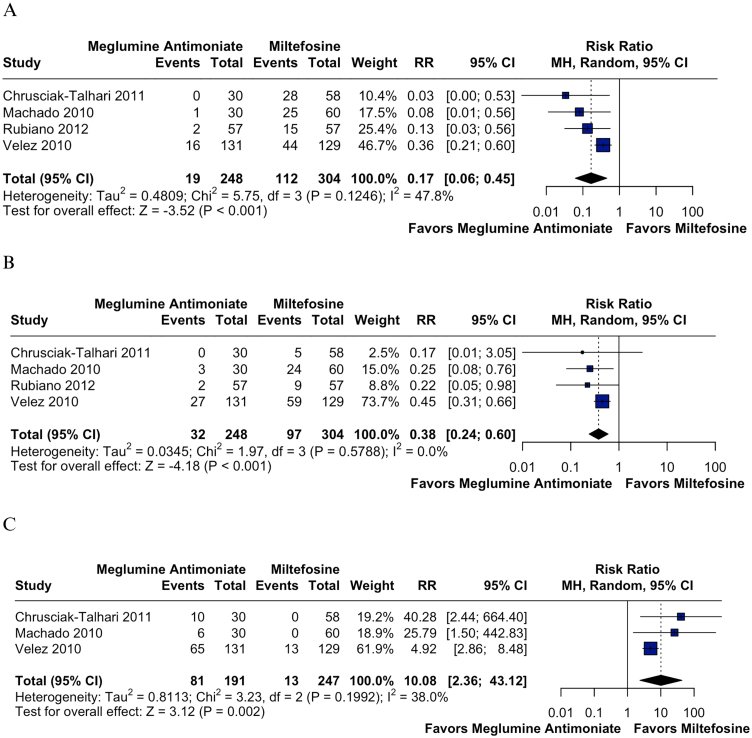
(A) Vomiting. (B) Nausea. (C) Arthralgia.

Gastrointestinal adverse events were consistently more frequent in the miltefosine group. Vomiting and nausea were assessed in four studies involving 552 patients, showing significantly lower risks with meglumine antimoniate: vomiting (RR = 0.17; 95% CI: 0.06–0.45; p < 0.001; *I*^2^ = 47.8%; Fig. 6A) and nausea (RR = 0.38; 95% CI: 0.24–0.60; p < 0.001; *I*^2^ = 0%; Fig. 6B).^22^, ^23^, ^26^, ^29^ Abdominal pain was evaluated in three studies including 464 patients, and also occurred significantly less often with meglumine antimoniate (RR = 0.33; 95% CI: 0.13–0.86; p = 0.023; *I*^2^ = 23.8%; Supplemental Fig. S15).^23^, ^26^, ^29^ Diarrhea was assessed in four studies with 552 patients, and although more frequent with miltefosine, the difference was not statistically significant (RR = 0.43; 95% CI: 0.18–1.07; p = 0.070; *I*^2^ = 0%; Supplemental Fig. S16).^22^, ^23^, ^26^, ^29^ The certainty of evidence for vomiting was rated moderate due to inconsistency (Supplemental Fig. S17).

In contrast, hepatic adverse events were more common with meglumine antimoniate, with significantly higher rates of ALT (RR = 2.31; 95% CI: 1.24–4.29; p = 0.008; *I*^2^ = 0%; Supplemental Fig. S18) and AST elevation (RR = 2.77; 95% CI: 1.39–5.52; p = 0.004; *I*^2^ = 0%; Supplemental Fig. S19), favoring miltefosine. These findings were based on two studies including 374 patients, with 186 treated with miltefosine and 188 with meglumine antimoniate.^26^, ^29^

Similarly, musculoskeletal and systemic symptoms were also more frequent with meglumine antimoniate. Arthralgia was assessed in three studies involving 438 patients and occurred significantly less often in the miltefosine group (RR = 10.08; 95% CI: 2.36–43.12; p = 0.002; *I*^2^ = 38.0%; Fig. 6C).^22^, ^23^, ^29^ Fever was evaluated in three studies, including 464 patients and also showed a significantly lower risk with miltefosine (RR = 2.98; 95% CI: 1.53–5.80; p = 0.001; *I*^2^ = 37.3%; Supplemental Fig. S20).^23^, ^26^, ^29^ Headache was assessed in two studies with a total of 204 patients and showed a non-significant trend in the same direction (RR = 1.57; 95% CI: 0.94–2.63; p = 0.086; *I*^2^ = 0%; Supplemental Fig. S21).^23^, ^26^ Arthralgia and fever were both rated as high certainty of evidence (Supplemental Fig. S17).

Leave-one-out and Baujat sensitivity analyses for vomiting, arthralgia, and fever are presented in the Supplemental material (Supplemental Figs. S22–S27).

### Quality and evidence assessment

**Fig. 7:**
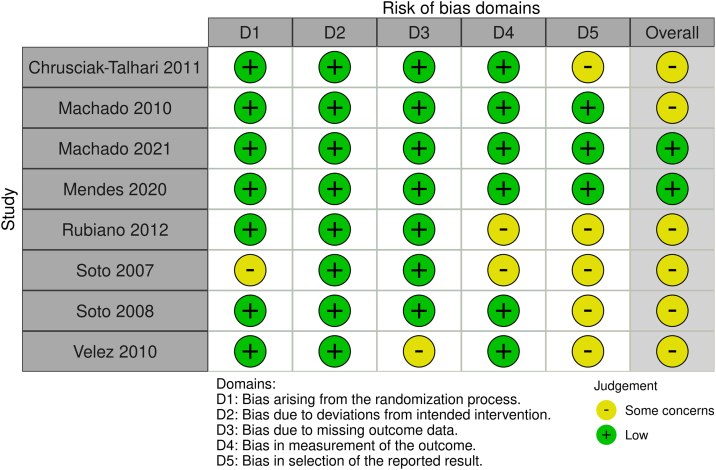
Risk of bias assessment of the included randomized controlled.

The individual appraisals of RCTs using the RoB-2 tool are illustrated in Fig. 7. Overall, most studies were rated as having “some concerns”, primarily due to bias in the selection of the reported result, as the majority of studies did not provide a trial protocol or statistical analysis plan.

## Discussion

This meta-analysis compared the efficacy and toxicity of meglumine antimoniate and miltefosine for CL, including 903 patients. The main findings were: (1) Miltefosine demonstrated superior cure rates at 2-months, with consistent effects across studies and high certainty of evidence; (2) No statistically significant difference was found at 1-, 3-, 4-, 6- or 12-months, although miltefosine showed a slight numerical advantage at 3- and 6-months; (3) In patients infected with *L. braziliensis*, miltefosine showed significantly higher cure rates at 2-months. However, no significant differences were observed at 3- or 6-months; (4) Miltefosine was associated with more gastrointestinal adverse events (e.g., nausea, vomiting), whereas meglumine antimoniate had higher rates of hepatic enzyme elevations, arthralgia, and fever. These findings suggest that while miltefosine offers some efficacy advantages and fewer systemic side effects, its gastrointestinal toxicity and potential for relapse should be carefully considered.

To contextualize these findings, it is important to review the therapeutic context. Miltefosine is recommended in endemic areas where injectable alternatives, such as pentavalent antimonials, liposomal amphotericin B, and paromomycin, present limitations. Despite its demonstrated efficacy, miltefosine has notable contraindications, including strict avoidance during pregnancy due to teratogenicity and its prolonged persistence in the body. It is also contraindicated in patients with severe renal or hepatic impairment.^30^, ^31^

Similarly, meglumine antimoniate requires careful consideration. Typically administered intravenously or intramuscularly over a similar treatment period to miltefosine, it demands cautious use in patients with preexisting cardiac, hepatic, or renal conditions because of risks such as antimony intolerance and arrhythmias. Like miltefosine, its use is contraindicated during pregnancy, and caution is recommended during breastfeeding despite limited clinical data.^32^

The pooled results showed superior early efficacy of miltefosine, particularly at the 2-month follow-up, aligning with findings from previous RCTs.In one study, 81.7% of patients receiving miltefosine achieved lesion cure at two months, compared to 60% in the meglumine antimoniate group.^23^ Similarly, another RCT reported apparent cure rates of 73.2% for miltefosine versus 60.7% for meglumine antimoniate at the same time point.^22^ These findings underscore miltefosine’s advantage in promoting a faster therapeutic response compared to traditional antimonial therapies.

However, while miltefosine demonstrates early efficacy benefits, differences in cure rates between miltefosine and meglumine antimoniate tend to diminish over time. No statistically significant differences were observed at 3-, 4-, 6-, or 12-months post-treatment, although miltefosine consistently maintained a slight numerical advantage at 3- and 6-months. Supporting these observations, a study on both Old World and New World CL by Iranpour et al. found that miltefosine was more effective than meglumine antimoniate at the 3-month follow-up, particularly when a high-weight study was excluded in sensitivity analyses. By the 6-month follow-up, pooled analyses revealed no significant difference in efficacy between the two treatments.^14^

In *L. braziliensis* infections, miltefosine achieved significantly higher cure rates at 2-months, but this advantage was not maintained at later follow-ups. Although miltefosine promotes faster initial healing, its long-term effectiveness appears comparable to that of meglumine antimoniate. Similar findings were reported by Soto et al. in 2007, who also evaluated *L. braziliensis* infections. In their study, miltefosine achieved higher cure rates than meglumine antimoniate at 2-months. By 4- and 6-months, however, the cure rates between the two treatments became comparable, with meglumine antimoniate slightly surpassing miltefosine at later time points.^28^ This pattern further supports the observation that miltefosine’s early benefit diminishes over time.

Although 2-month cure rates were reported in clinical trials, they reflect early treatment response rather than definitive cure. Olliaro et al., in a methodological guide for clinical trials in cutaneous leishmaniasis, propose a standardized framework in which outcomes are assessed at three key time points: 6–9 weeks for initial response, 3-months for initial cure, and 6–12 months for definitive cure, the latter being crucial to capture late relapses and ensure long-term efficacy.^33^ Complementing this, the World Health Organization considers the absence of clinical relapse at 6-months a reliable indicator of sustained cure, as relapses may occur several months after initial lesion healing.^34^ Therefore, while 2-month cure rates provide clinically relevant information on early lesion resolution, they should not be interpreted as definitive evidence of parasitological cure. Longer follow-up, preferably up to 6- or 12-months, is essential for reliable efficacy assessment.^34^

In terms of failure rates, this analysis showed no statistically significant difference in cure failure at 6-months. However, the analysis revealed considerable heterogeneity and sensitivity of the pooled estimate, largely driven by the influence of a single study. When this study was excluded in sensitivity analysis, the results shifted in favor of meglumine antimoniate, suggesting that the long-term efficacy of miltefosine may be less consistent in certain settings. Similar concerns have been raised in previous studies. One pilot study reported that although all patients showed initial clinical improvement after a 28-day course of miltefosine, only 48.7% achieved complete cure at 6-months, with a relapse rate of 32.3%.^35^ These findings align with the trend observed in the meta-analysis, highlighting potential limitations of miltefosine in sustaining long-term outcomes despite its early efficacy. Nonetheless, other studies have reported contrasting results. A separate cohort study found that pentavalent antimonials were associated with higher relapse rates than miltefosine.^36^ These discrepancies emphasize the need for further high-quality studies to better understand factors affecting long-term treatment success and relapse.

Safety profiles also differed between treatments. Meglumine antimoniate was associated with a higher incidence of systemic and musculoskeletal adverse events, including significantly increased rates of arthralgia and fever, both supported by high-certainty evidence, as well as elevations in hepatic enzymes (ALT and AST). Similarly, studies on systemic meglumine antimoniate treatment have highlighted its broad spectrum of side effects, ranging from mild symptoms, such as muscle and joint pain, gastrointestinal disturbances, fatigue, fever, and skin reactions, to severe, life-threatening complications like cardiovascular abnormalities and liver or pancreatic dysfunction.^2^, ^37^ The present study also found that miltefosine was linked to a higher incidence of gastrointestinal issues, particularly nausea and vomiting, consistent with a meta-analysis on interventions for CL and mucocutaneous leishmaniasis, which reported higher rates of nausea and vomiting with miltefosine compared to meglumine antimoniate.^16^

Beyond efficacy and safety, cost and accessibility are also critical considerations. A recent study compared the costs of meglumine antimoniate and miltefosine with caregiver directly observed therapy. It found miltefosine more cost-effective for patients and society due to lower travel and lodging costs compared to meglumine antimoniate. The study concluded that miltefosine is cost-saving for patients and society, with a minimal increase in government expenses.^38^ Given miltefosine’s early efficacy and safety, these cost benefits make a strong case for its wider use, especially in resource-limited areas where access to treatment is a challenge.

This meta-analysis has several important strengths. It includes eight RCTs conducted across diverse regions in Latin America, focusing exclusively on New World cutaneous leishmaniasis to ensure geographic and clinical relevance. The methodology adhered strictly to PRISMA guidelines, ensuring a transparent and rigorous selection and appraisal process. Risk of bias was systematically evaluated using the Cochrane RoB-2 tool, and the certainty of evidence was assessed with the GRADE approach. To address variability and strengthen the robustness of findings, leave-one-out sensitivity analyses and Baujat plots were employed to identify sources of heterogeneity and assess the influence of individual studies.

Nonetheless, some limitations should be considered. High heterogeneity was observed in certain outcomes, which may reduce the precision and generalizability of pooled estimates. Several studies had relatively short follow-up periods, potentially underestimating late relapses or long-term adverse effects. In addition, small sample sizes in some comparisons limited the power to detect rare adverse events and may have contributed to imprecision in safety outcomes. Finally, despite these efforts to standardize data collection, variations in treatment protocols, patient populations, and outcome definitions across studies could have influenced the results.

Notwithstanding these challenges, this systematic review and meta-analysis offer a comprehensive overview of the most robust evidence regarding the efficacy and safety of miltefosine compared to meglumine antimoniate for the treatment of CL in the New World.

## Conclusion

This systematic review and meta-analysis demonstrate that miltefosine offers superior early treatment response compared to meglumine antimoniate for ACL, particularly at two months. However, this advantage diminishes over time, with cure rates becoming comparable at later follow-ups. Miltefosine was linked to more gastrointestinal side effects, while meglumine antimoniate had a higher risk of hepatic and systemic adverse events. The certainty of evidence, as assessed by GRADE, ranged from high for early efficacy and certain safety outcomes to very low for long-term efficacy and cure failure, primarily due to inconsistency and imprecision across studies. This variability limits confidence in sustained treatment effects over time. Considering its early efficacy, safety profile, and cost-effectiveness, miltefosine remains a valuable treatment option, particularly in resource-limited settings where oral administration is advantageous. Nonetheless, the inconsistent durability of cure and limited long-term data highlight the need for further high-quality studies with extended follow-up.

## ORCID ID

Ana Carolina Putini Vieira: 0009-0001-8714-2962

Fernanda Cronemberger Lins: 0009-0008-3350-7787

Arianne Costa Baquião: 0000-0003-3921-9256

## Financial support

None declared.

## Authors’ contributions

Ana Carolina Putini Vieira: The study concept and design; critical review of literature; data collection, analysis and interpretation; preparation and writing of the manuscript; final approval of the final version of the manuscript.

Fernanda Cronemberger Lins: Critical review of literature; data collection, analysis and interpretation; preparation and writing of the manuscript; final approval of the final version of the manuscript.

Arianne Costa Baquião: The study concept and design; preparation and writing of the manuscript; effective participation in research orientation; manuscript critical review; final approval of the final version of the manuscript.

## Research data availability

The entire dataset supporting the results of this study was published in this article.

## Conflicts of interest

None declared.
