# Supplementary material for: Comparison of meglumine antimoniate versus miltefosine in the treatment of new world cutaneous leishmaniasis: a systematic review and meta-analysis
Source: An Bras Dermatol. 2026 Jan 8;101(1):501253. doi: 10.1016/j.abd.2025.501253 (PMC12813531; doi:10.1016/j.abd.2025.501253)
Supplement: Supplementary file 1 [file mmc1.docx]

**Comparison of meglumine antimoniate versus miltefosine in the treatment of new world cutaneous leishmaniasis: a systematic review and meta-analysis**

**Journal:** Anais Brasileiros de Dermatologia

**Contents:**

1. Supplemental figure 1. Cure rates at 1 month post-treatment
2. Supplemental figure 2. Baujat analysis Cure rates at 1 month post-treatment
3. Supplemental figure 3. Leave-one-out Cure rates at 3 months post treatment
4. Supplemental figure 4. Baujat analysis Cure rates at 3 months post treatment
5. Supplemental figure 5. Cure rates at 4 months post-treatment
6. Supplemental figure 6. Leave-one-out Cure rates at 6 months post treatment
7. Supplemental figure 7. Baujat analysis Cure rates at 6 months post treatment
8. Supplemental figure 8. Cure rates at 12 months post-treatment
9. Supplemental figure 9. Baujat analysis Cure rates at 12 month post-treatment
10. Supplemental figure 10. Baujat Analysis Cure Rates at 3 months in *L. braziliensis* infection
11. Supplemental figure 11. Leave-one-out Cure Rates at 6 months in *L. braziliensis* infection
12. Supplemental figure 12. Baujat analysis Cure Rates at 6 months in *L. braziliensis* infection
13. Supplemental figure 13. Leave-one-out Cure Failure at 6 months
14. Supplemental figure 14. Baujat analysis Cure Failure at 6 months
15. Supplemental figure 15. Abdominal pain
16. Supplemental figure 16. Diarrhea
17. Supplemental figure 17. GRADE assessment - Adverse Events
18. Supplemental figure 18. Alanine Aminotransferase (ALT)
19. Supplemental figure 19. Aspartate Aminotransferase (AST)
20. Supplemental figure 20. Fever
21. Supplemental figure 21. Headache
22. Supplemental figure 22. Leave-one-out Vomiting
23. Supplemental figure 23. Baujat analysis Vomiting
24. Supplemental figure 24. Leave-one-out Arthralgia
25. Supplemental figure 25. Baujat Analysis Arthralgia
26. Supplemental figure 26. Leave-one-out Fever
27. Supplemental figure 27. Baujat analysis Fever

Supplemental Figure 1 Cure rates at 1 month post-treatment


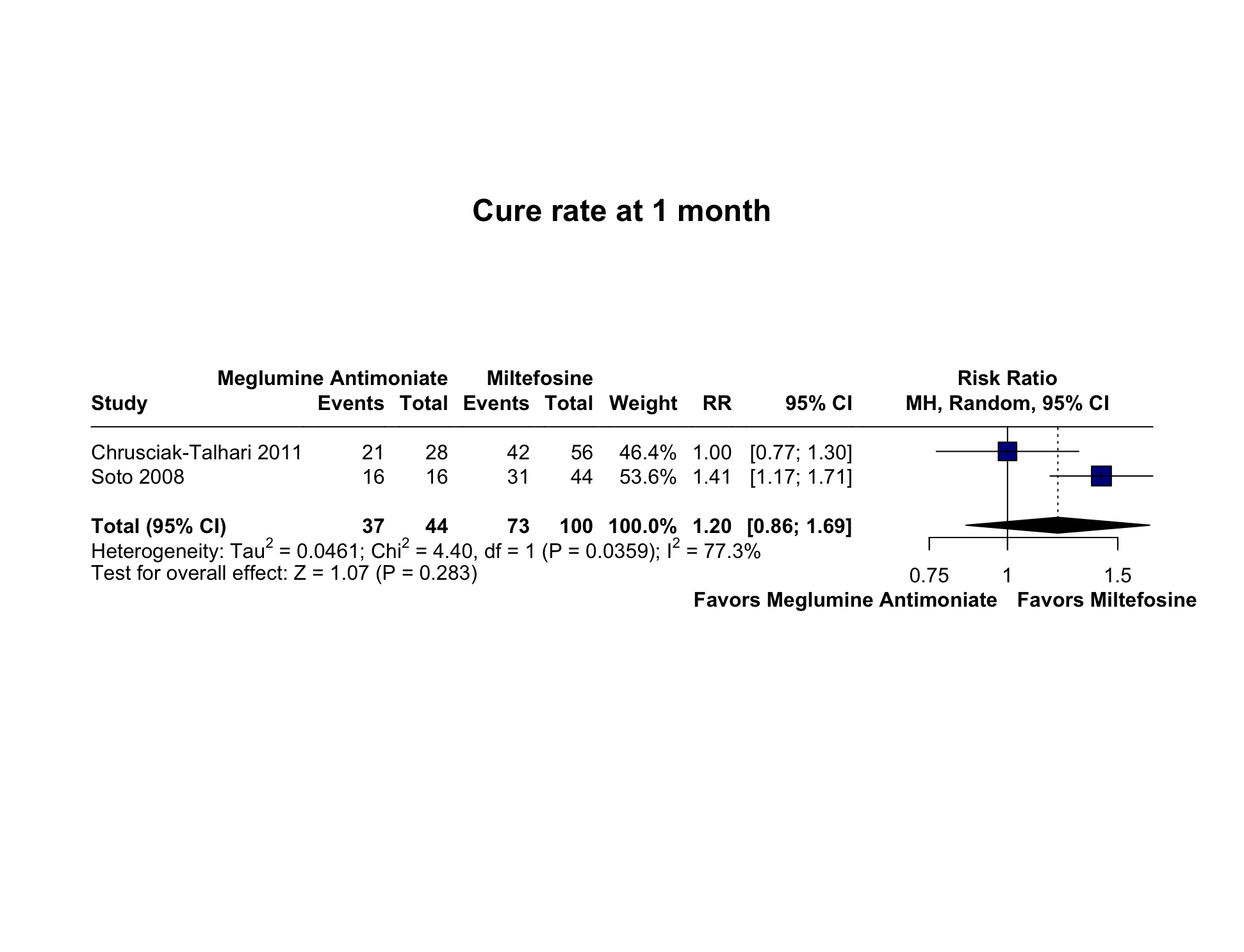


Two studies were included in the analysis of cure rate at 1 month.^1,6^ When pooling the results, no statistically significant difference was observed between miltefosine and meglumine antimoniate (RR 1.20; 95% CI 0.86–1.69; P = 0.283; I² = 77.3%). The prediction interval was not reported. Despite one study (Soto et al., 2008) favoring miltefosine (RR 1.41; 95% CI 1.17–1.71), overall heterogeneity was high (Chi² = 4.40; P = 0.0359), and the pooled estimate did not reach statistical significance.

Supplemental Figure 2. Baujat analysis Cure rates at 1 month post-treatment


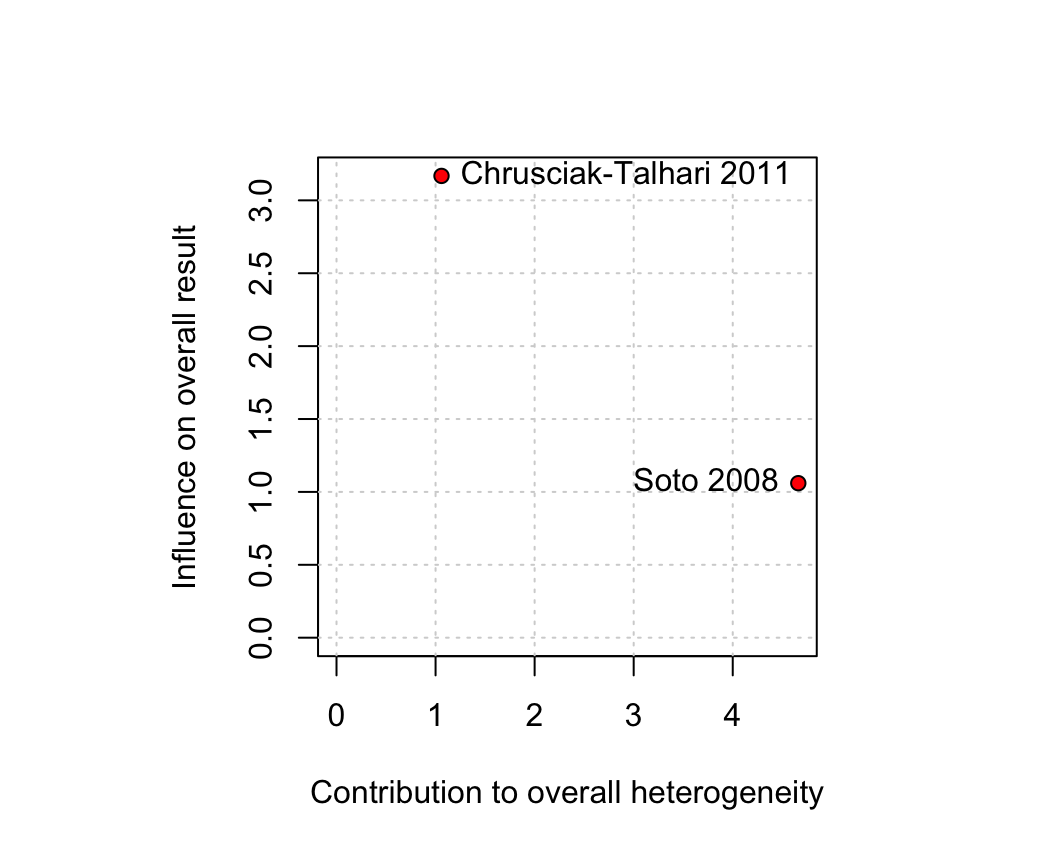


In the Baujat plot for the 1-month cure rate outcome, Chrusciak-Talhari et al. 2011 contributed the most to the influence on the overall result, while Soto et al. 2008 contributed the most to the overall heterogeneity.^1,6^ Both studies showed moderate influence and heterogeneity contributions, suggesting that no single study disproportionately affected the pooled estimate.

Supplemental Figure 3 Leave-one-out Cure rates at 3 months post treatment


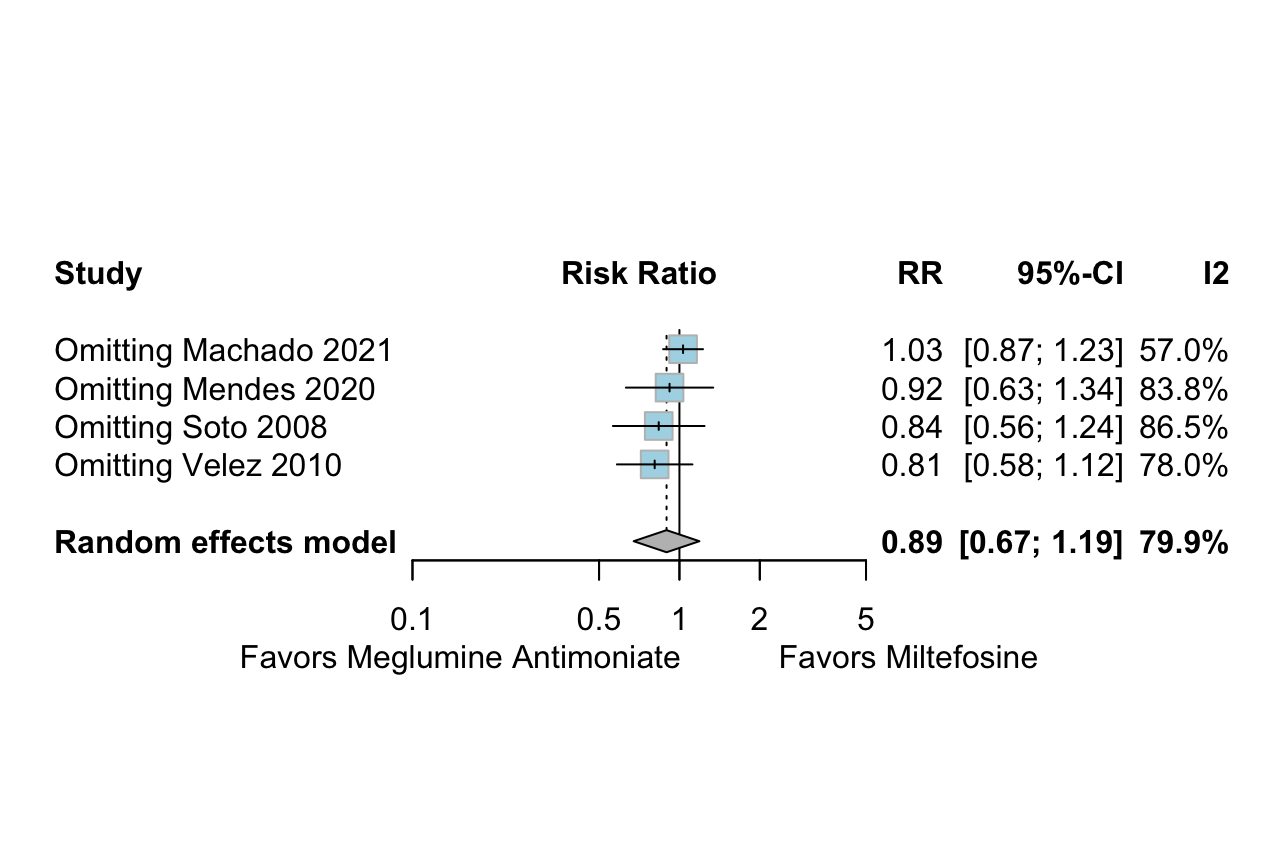


For the outcome of cure rate at 3 months, leave-one-out analysis showed a reduction in heterogeneity when omitting individual studies. The exclusion of Machado et al. 2021 reduced I² from 79.9% to 57.0%, with a pooled RR of 1.03 (95% CI 0.87–1.23).^3^ Omitting Mendes et al. 2020, Soto et al. 2008, and Velez et al. 2010 also decreased heterogeneity to varying extents (I² = 83.8%, 86.5%, and 78.0%, respectively), but the overall effect remained non-significant (RR range 0.81–0.92).^4,6,8^ The overall pooled estimate was RR 0.89 (95% CI 0.67–1.19).

Supplemental Figure 4 Baujat analysis Cure rates at 3 months post treatment


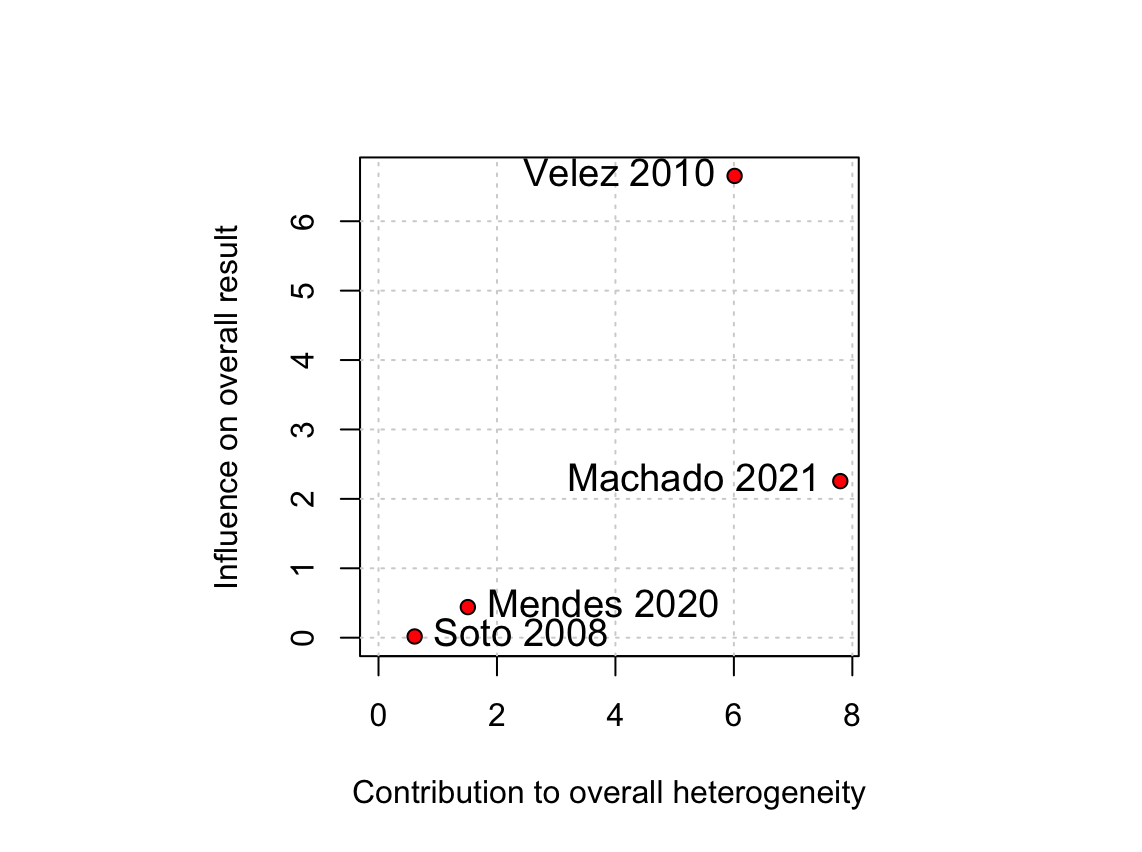


The Baujat plot for the cure rate at 3 months identified Velez et al. 2010 and Machado et al. 2021 as the primary contributors to both heterogeneity and influence on the overall effect estimate.^3,8^

Supplemental Figure 5 Cure rates at 4 months post-treatment


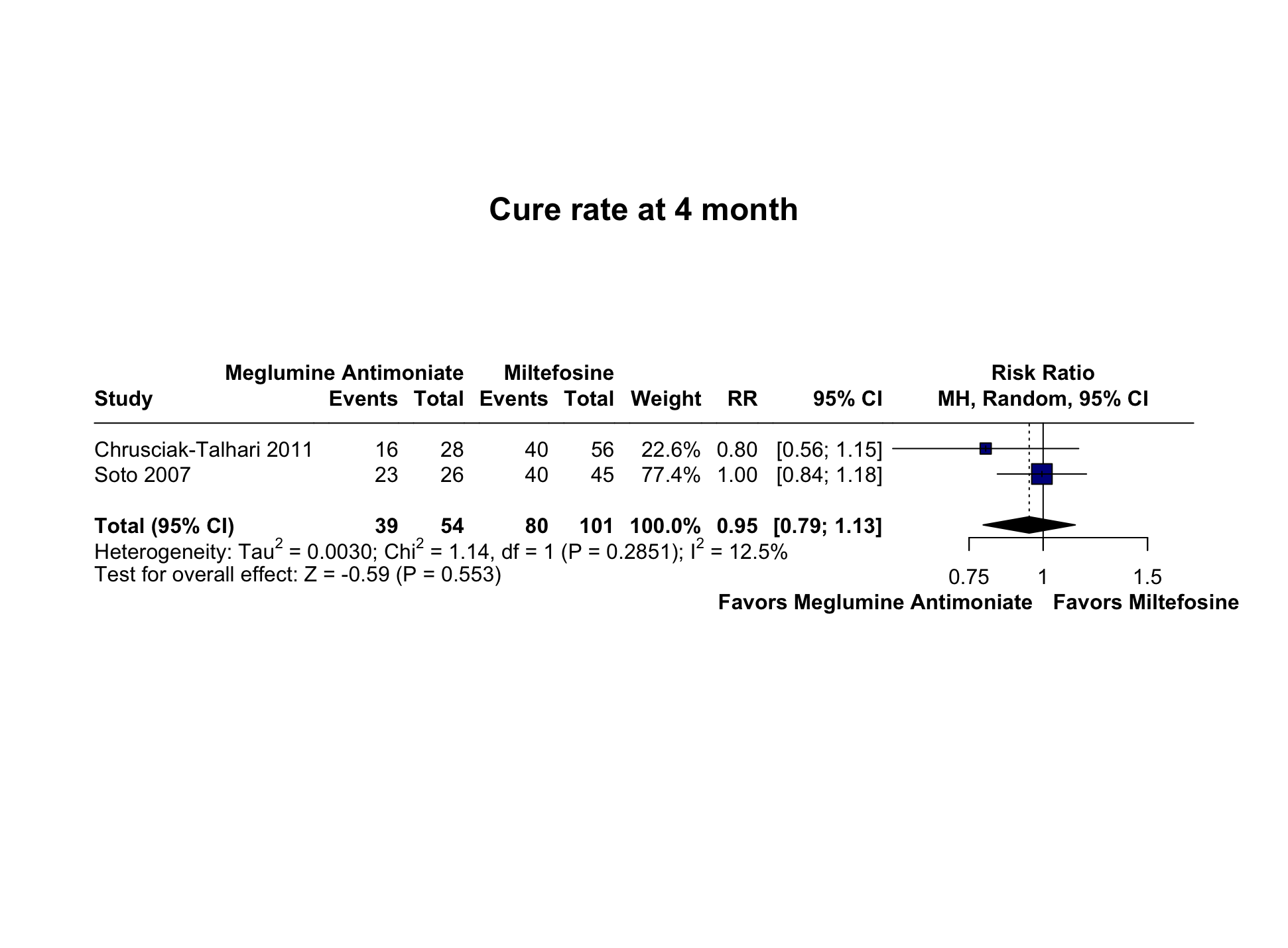


Two studies reported cure rates at 4 months.^1,7^ The pooled analysis showed no statistically significant difference between miltefosine and meglumine antimoniate (RR 0.95; 95% CI 0.79–1.13; p = 0.553; I² = 12.5%). Individually, Chrusciak-Talhari et al. 2011 reported a slightly lower cure rate with miltefosine (RR 0.80; 95% CI 0.56–1.15), while Soto et al. 2007 found no difference (RR 1.00; 95% CI 0.84–1.18). Heterogeneity was low (I² = 12.5%), indicating consistent findings across the studies.

Supplemental Figure 6 Leave-one-out Cure rates at 6 months post treatment


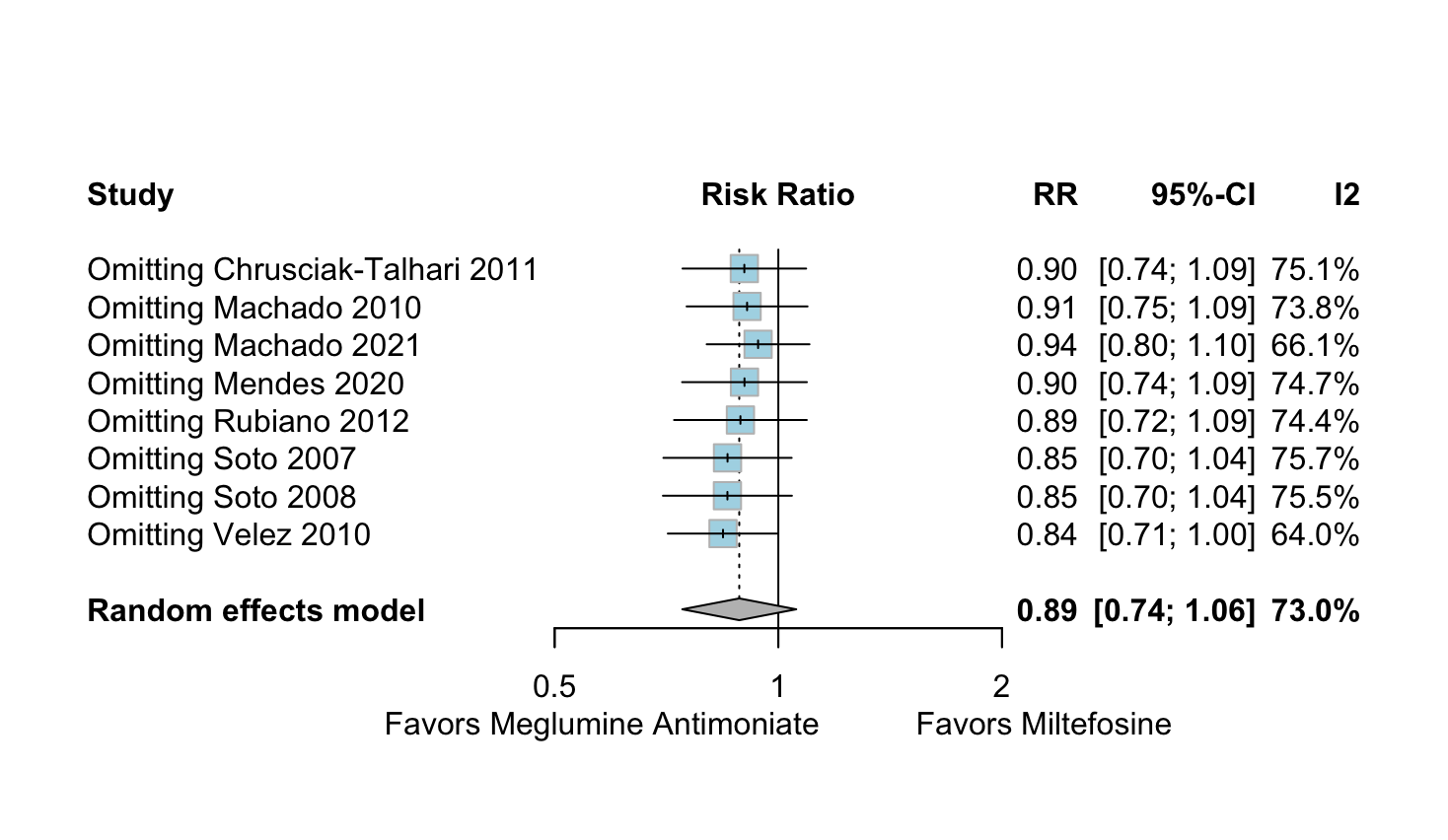


The pooled risk ratio remained stable (RR 0.89; 95% CI 0.74–1.06; I² = 73.0%) when each study was omitted in turn. The largest change in heterogeneity occurred when Velez et al. 2010 was excluded, reducing I² from 73.0% to 64.0% and yielding a slightly lower RR (0.84; 95% CI 0.71–1.00), suggesting this study contributed most to heterogeneity and the pooled estimate.^8^ However, no omission led to a statistically significant change in the overall result.

Supplemental Figure 7 Baujat analysis Cure rates at 6 months post treatment


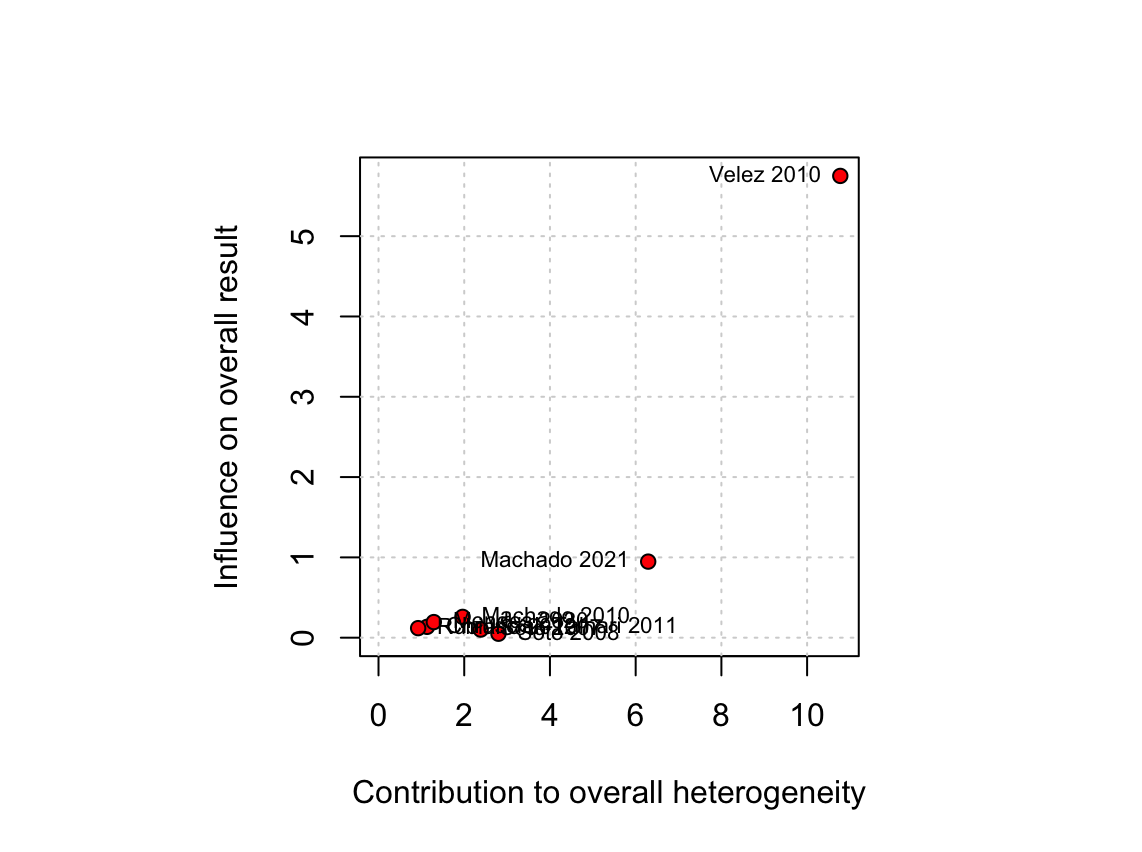


The Baujat plot for the cure rate at 6 months revealed that the study by Vélez et al. 2010 contributed the most to both overall heterogeneity and influence on the pooled result. Machado et al. 2021 also showed moderate influence, while the remaining studies had minimal contribution to heterogeneity and little impact on the overall effect estimate.^3,8^ This suggests that Vélez et al. 2010 was the primary driver of heterogeneity and variability in the pooled analysis for this outcome.^8^

Supplemental Figure 8 Cure rates at 12 months post-treatment


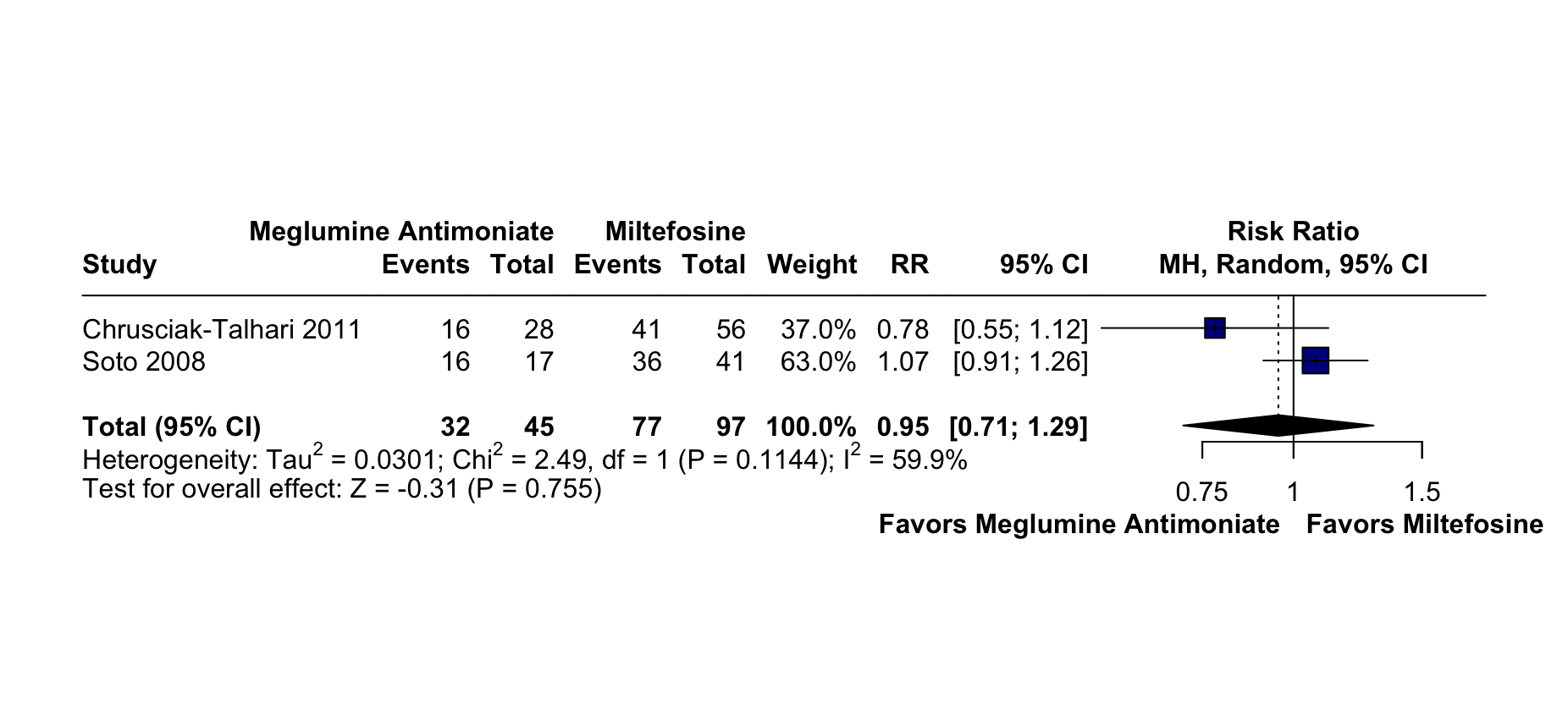


Two studies contributed data on cure rates at 12 months. The pooled analysis showed no statistically significant difference between miltefosine and meglumine antimoniate (RR 0.95; 95% CI 0.71–1.29; P = 0.755). Heterogeneity was moderate (I² = 59.9%). Individually, Soto et al. 2008 reported a slightly higher cure rate for miltefosine (RR 1.07; 95% CI 0.91–1.26), while Chrusciak-Talhari et al. 2011 favored meglumine antimoniate numerically (RR 0.78; 95% CI 0.55–1.12), though neither comparison reached statistical significance.^1,7^

Supplemental Figure 9. Baujat analysis Cure rates at 12 month post-treatment


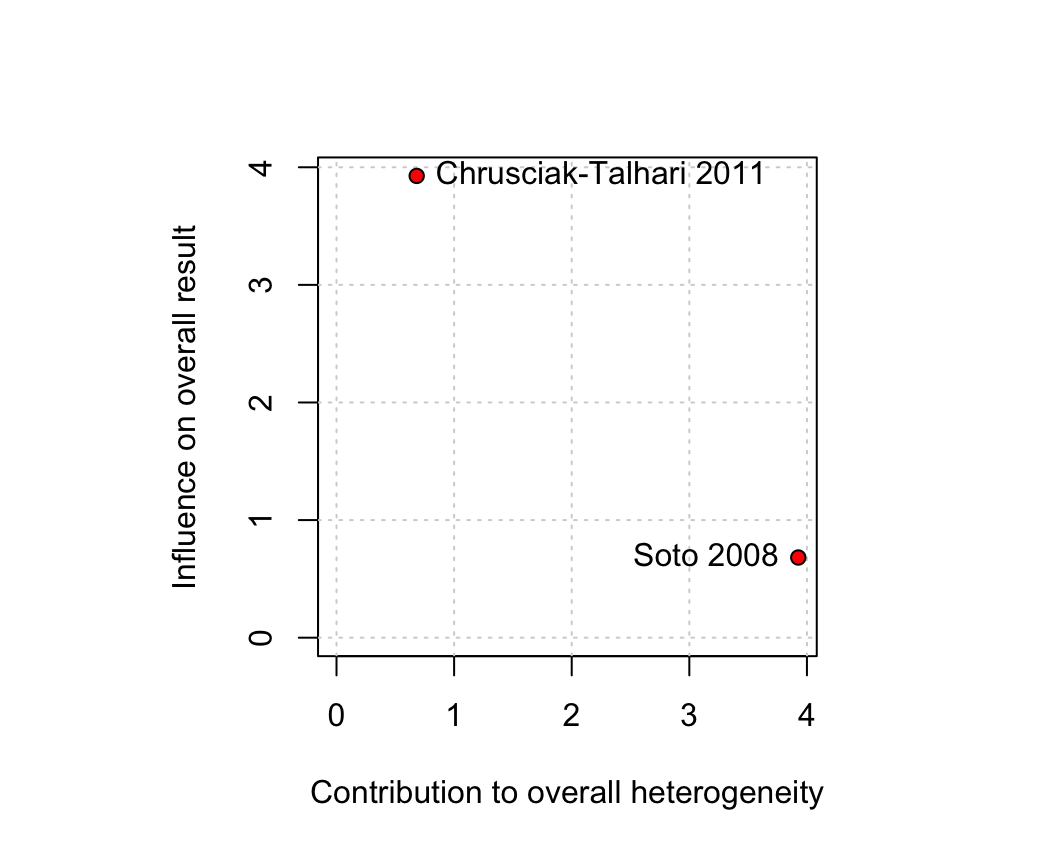


In the Baujat plot for cure rates at 12 months, the Chrusciak-Talhari et al. 2011 study demonstrated the greatest influence on the overall pooled result, while Soto et al. 2008 contributed the most to overall heterogeneity.^1,6^ Despite these contributions, neither study appears to be a clear outlier, although they are the primary drivers of both heterogeneity and influence in this time point.

Supplemental Figure 10 Baujat Analysis Cure Rates at 3 months in L. braziliensis infection


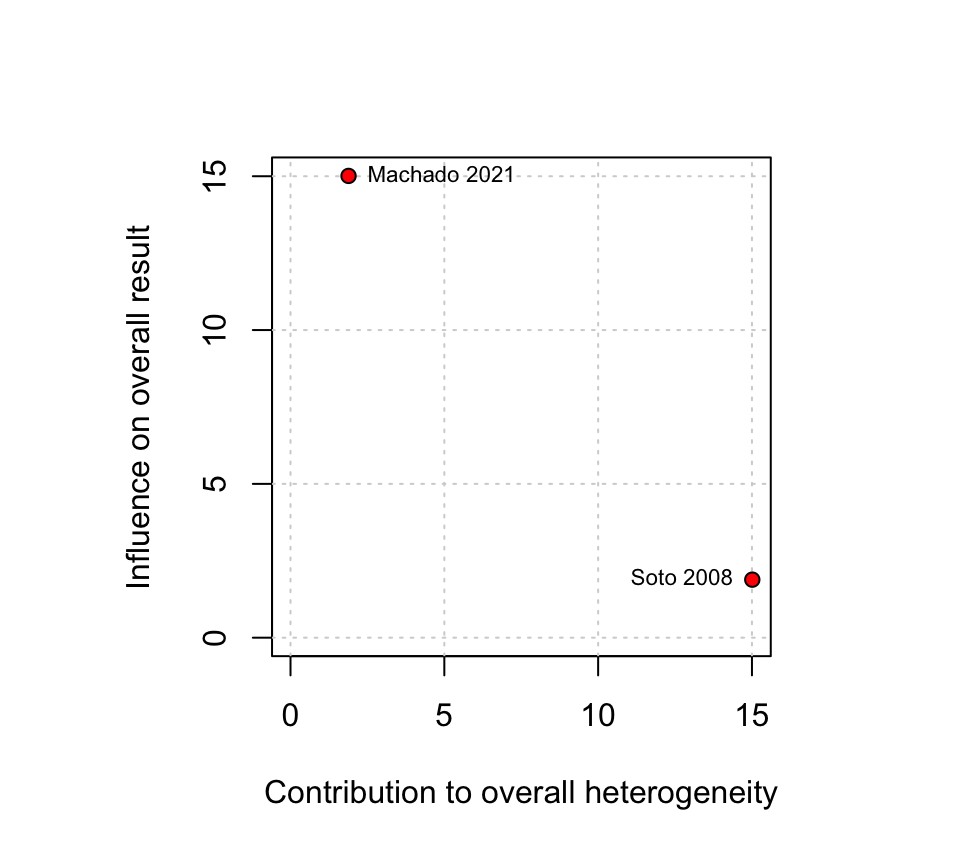


The Baujat plot indicated that Machado et al. 2021 contributed the most to the influence on the overall result, while Soto et al. 2008 contributed most to the overall heterogeneity. These two studies were the primary drivers of inconsistency in the pooled estimate for the 3-month cure rate in *L. braziliensis* infections.^3,6^

Supplemental Figure 11 Leave-one-out Cure Rates at 6 months in L. braziliensis infection


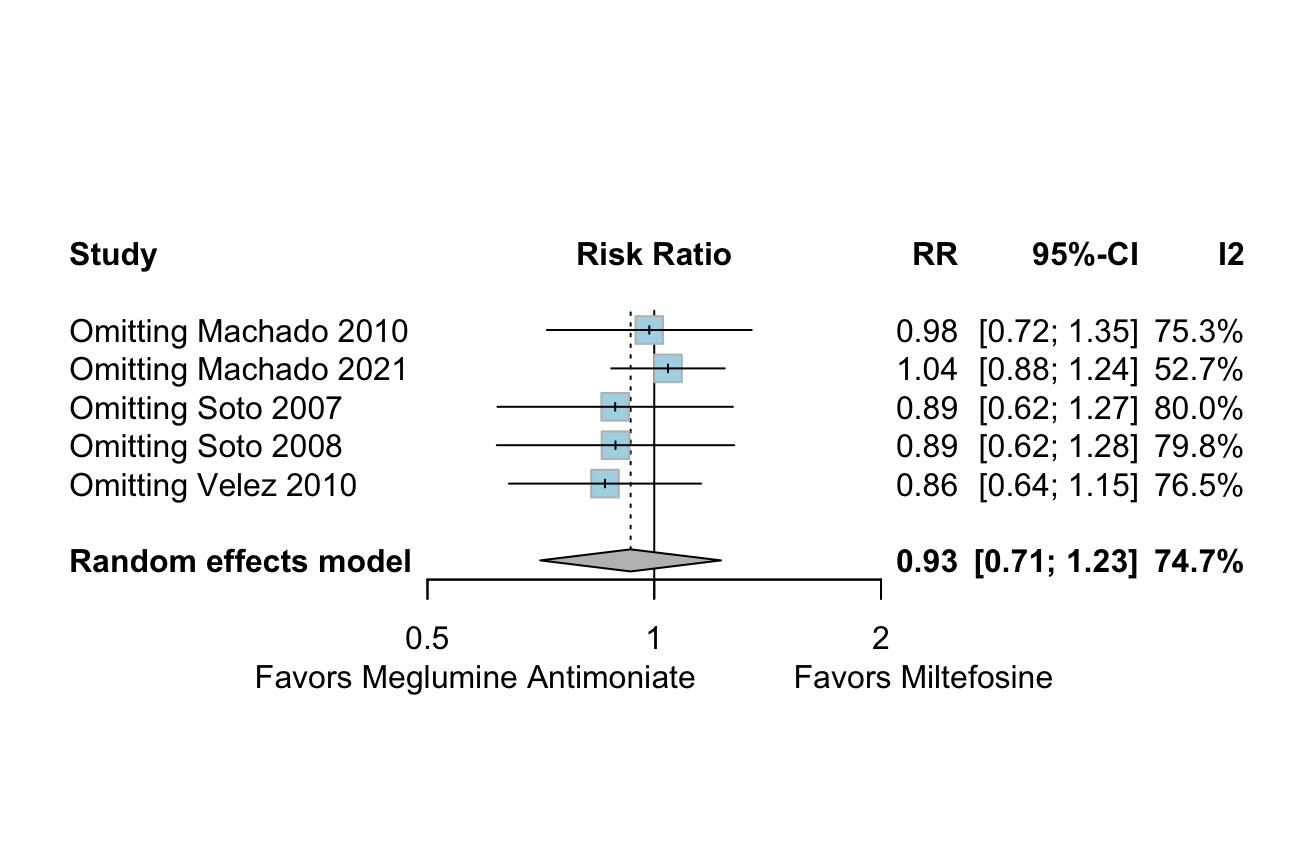


For the 6-month cure rate in *L. braziliensis* infections, leave-one-out sensitivity analysis revealed minimal changes in the pooled effect size (RR 0.93; 95% CI 0.71–1.23). The exclusion of Machado et al. 2021 notably reduced heterogeneity from I² = 74.7% to I² = 52.7%,^3^ but the overall interpretation remained unchanged, indicating that no single study disproportionately affected the robustness of the pooled estimate.

Supplemental Figure 12 Baujat analysis Cure Rates at 6 months in L. braziliensis infection


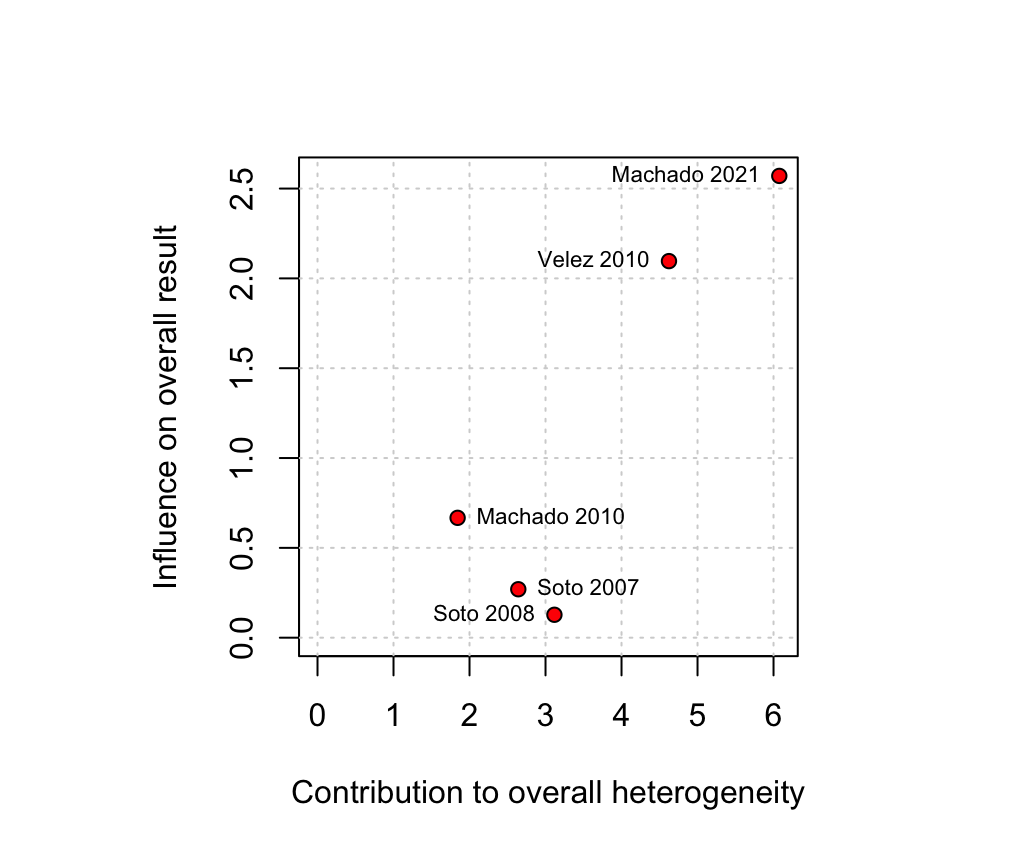


The Baujat plot indicated that Machado et al, 2021 and Velez et al. 2010 contributed the most to both overall heterogeneity and influence on the pooled effect.^3,8^

Supplemental Figure 13 Leave-one-out Cure Failure at 6 months


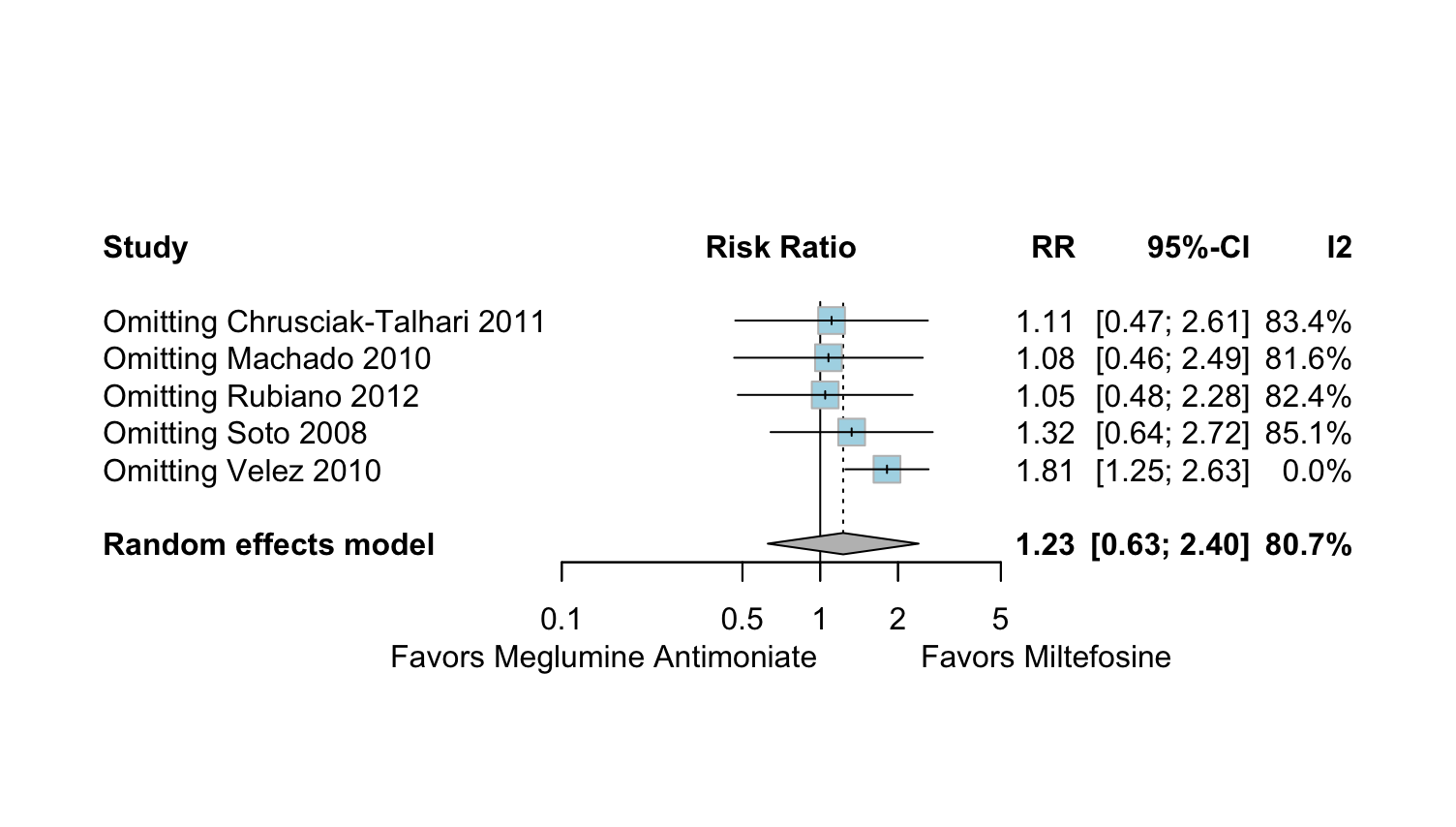


For cure failure at 6 months, omitting Velez et al. 2010 substantially reduced heterogeneity from I² = 80.7% to 0% and increased the pooled RR to 1.81 (95% CI: 1.25–2.63), indicating this study had a major influence on both heterogeneity and the overall effect estimate.^8^

Supplementary Figure 15 Baujat analysis Cure Failure at 6 months


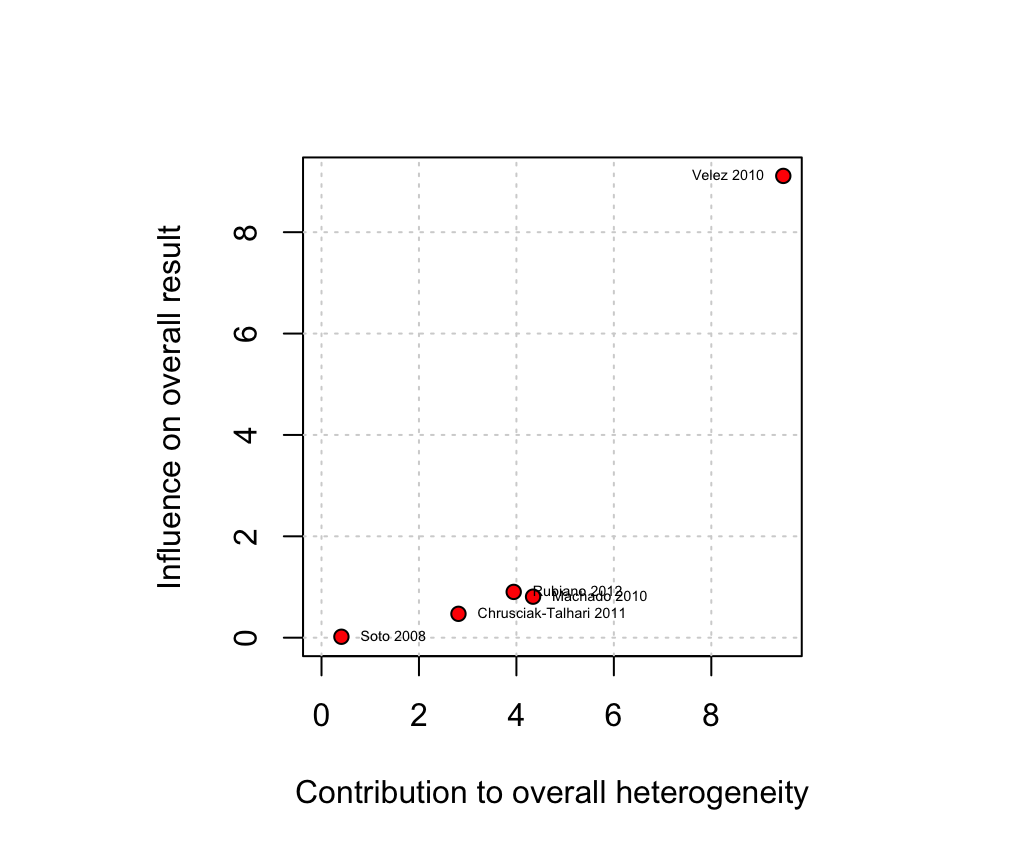


The Baujat plot for cure failure at 6 months indicated that Velez et al. 2010 contributed most to both overall heterogeneity and influence on the pooled result.^8^

Supplemental Figure 15 Abdominal pain


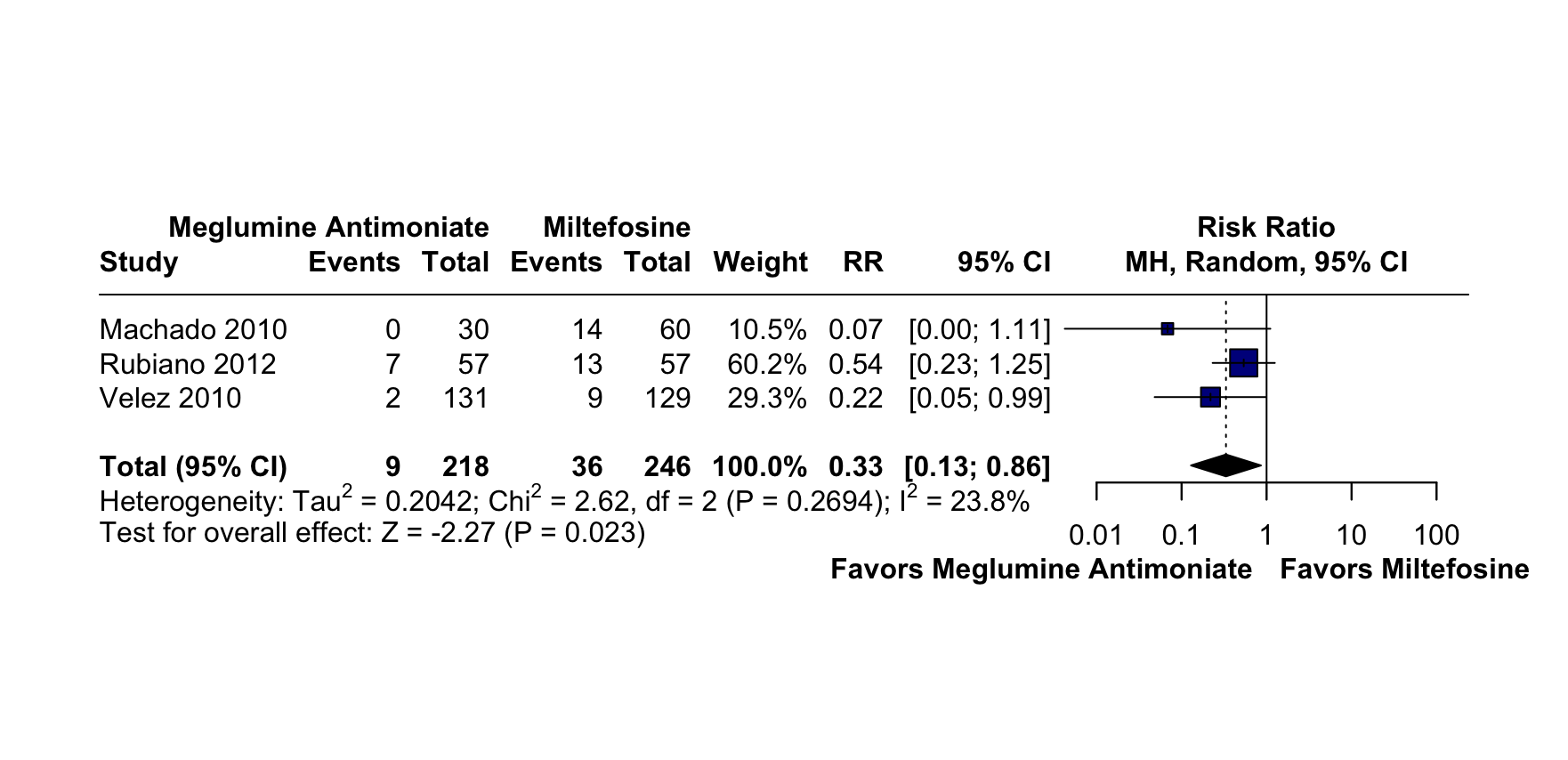


Three studies were included in the analysis of abdominal pain.^2,5,8^ The pooled results showed that abdominal pain was significantly less frequent in the meglumine antimoniate group compared to miltefosine (RR 0.33; 95% CI 0.13–0.86; P = 0.023; I² = 23.8%). The test for heterogeneity was not statistically significant (Chi² = 2.62, P = 0.2694), and the overall heterogeneity was low. These findings indicate a lower risk of abdominal pain with meglumine antimoniate treatment.

Supplemental Figure 16 Diarrhea


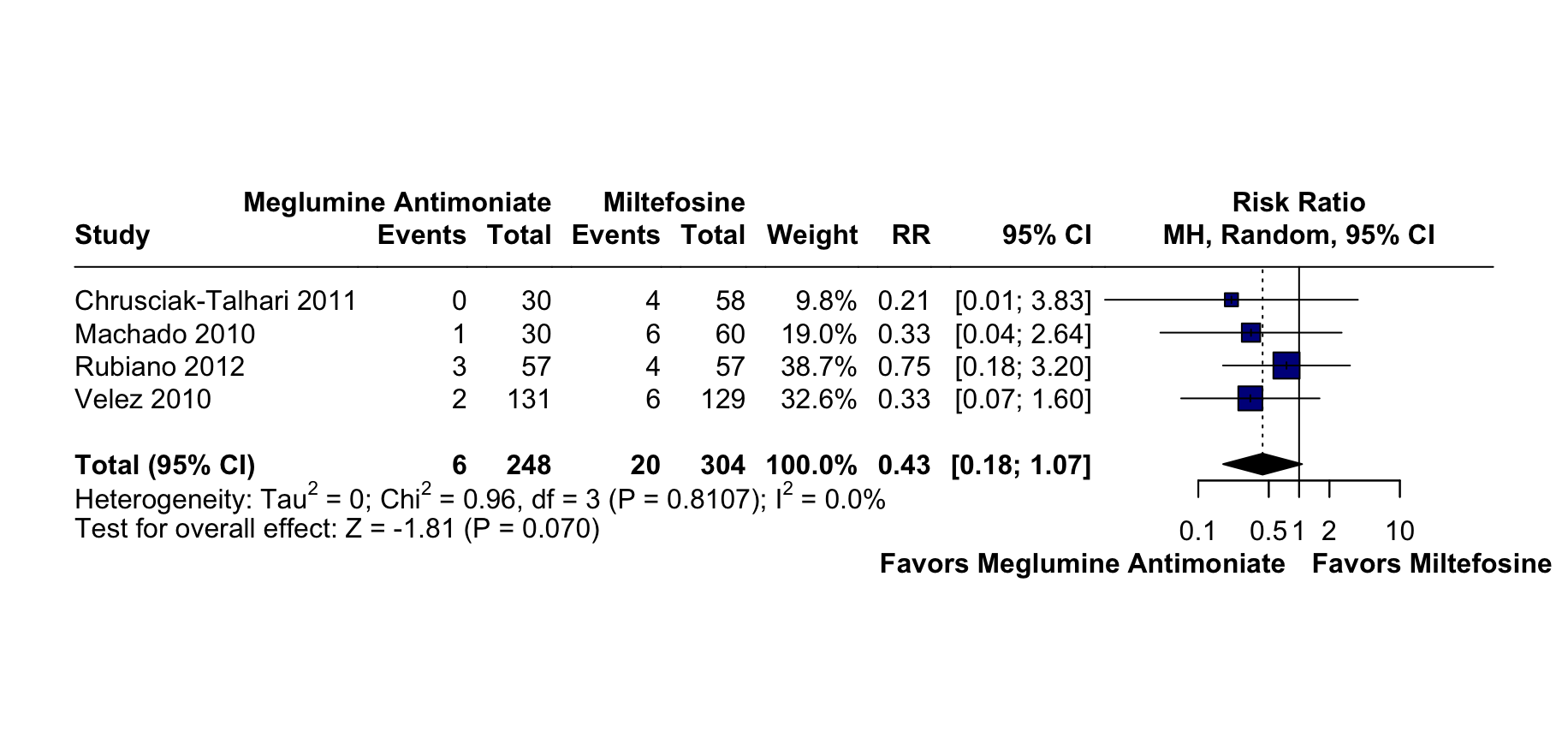


Four studies were included in the analysis of diarrhea.^1,2,5,8^ The pooled results showed no statistically significant difference in the risk of diarrhea between the meglumine antimoniate and miltefosine groups (RR 0.43; 95% CI 0.18–1.07; P = 0.070; I² = 0%). The heterogeneity among studies was negligible (Chi² = 0.96; P = 0.8107).

Supplemental Figure 17. GRADE assessment - Adverse Events


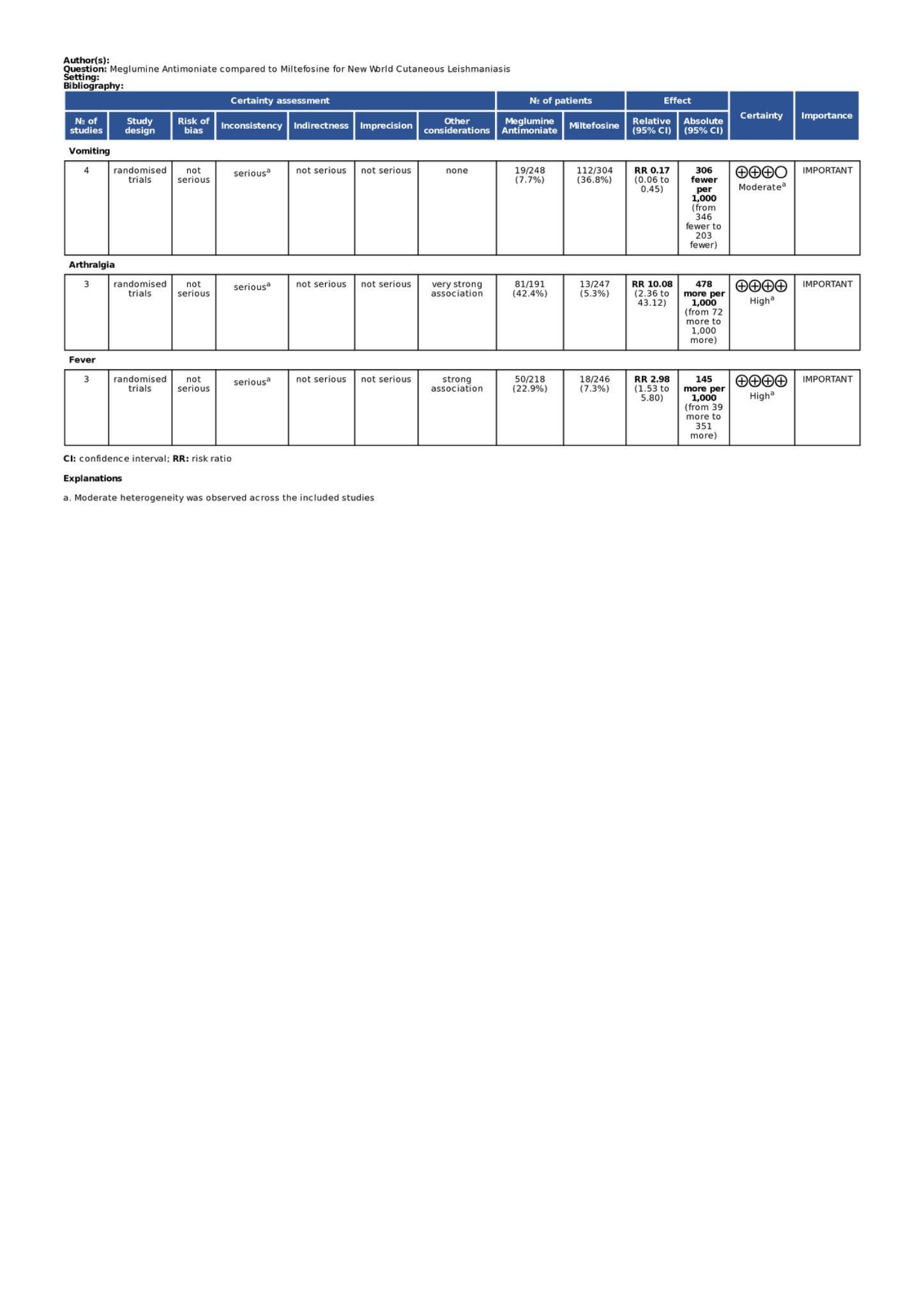


Supplementary Figure 19 Alanine Aminotransferase (ALT)


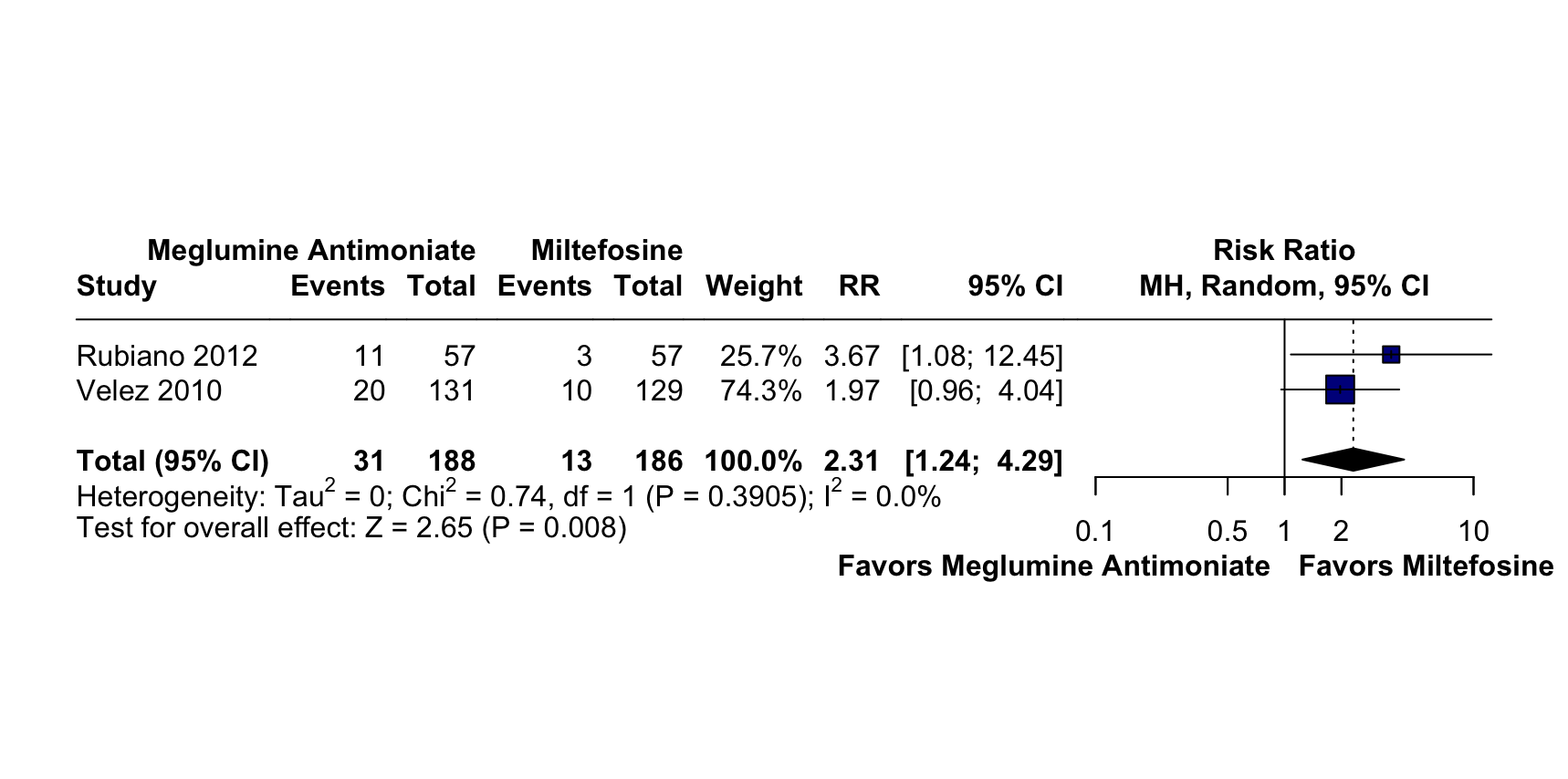


Two studies reported data on ALT elevation.^5,8^

Supplementary Figure 20 Aspartate Aminotransferase (AST)


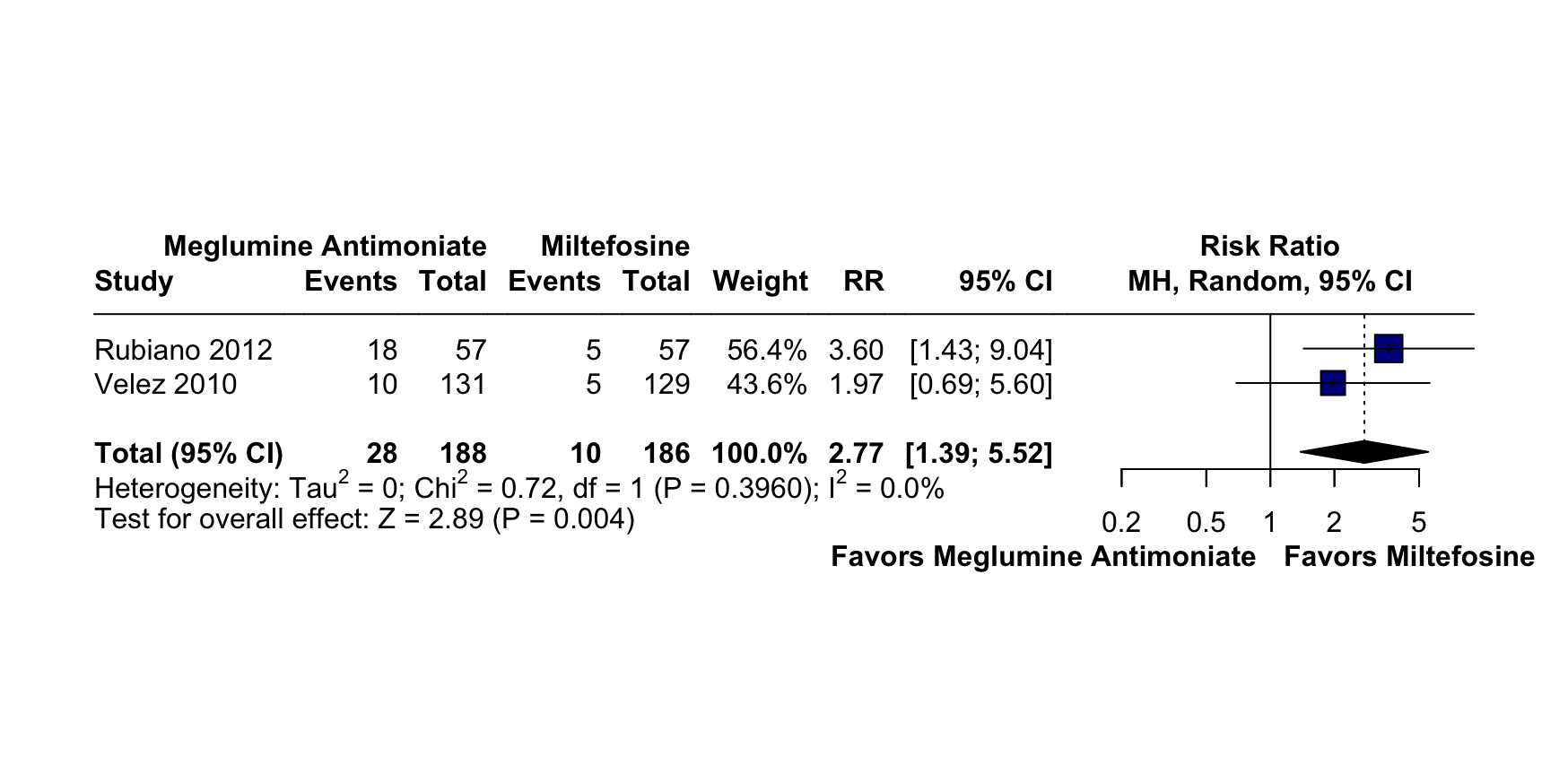


Two studies reported data on ALT elevation.^5,8^

Supplemental Figure 20 Fever


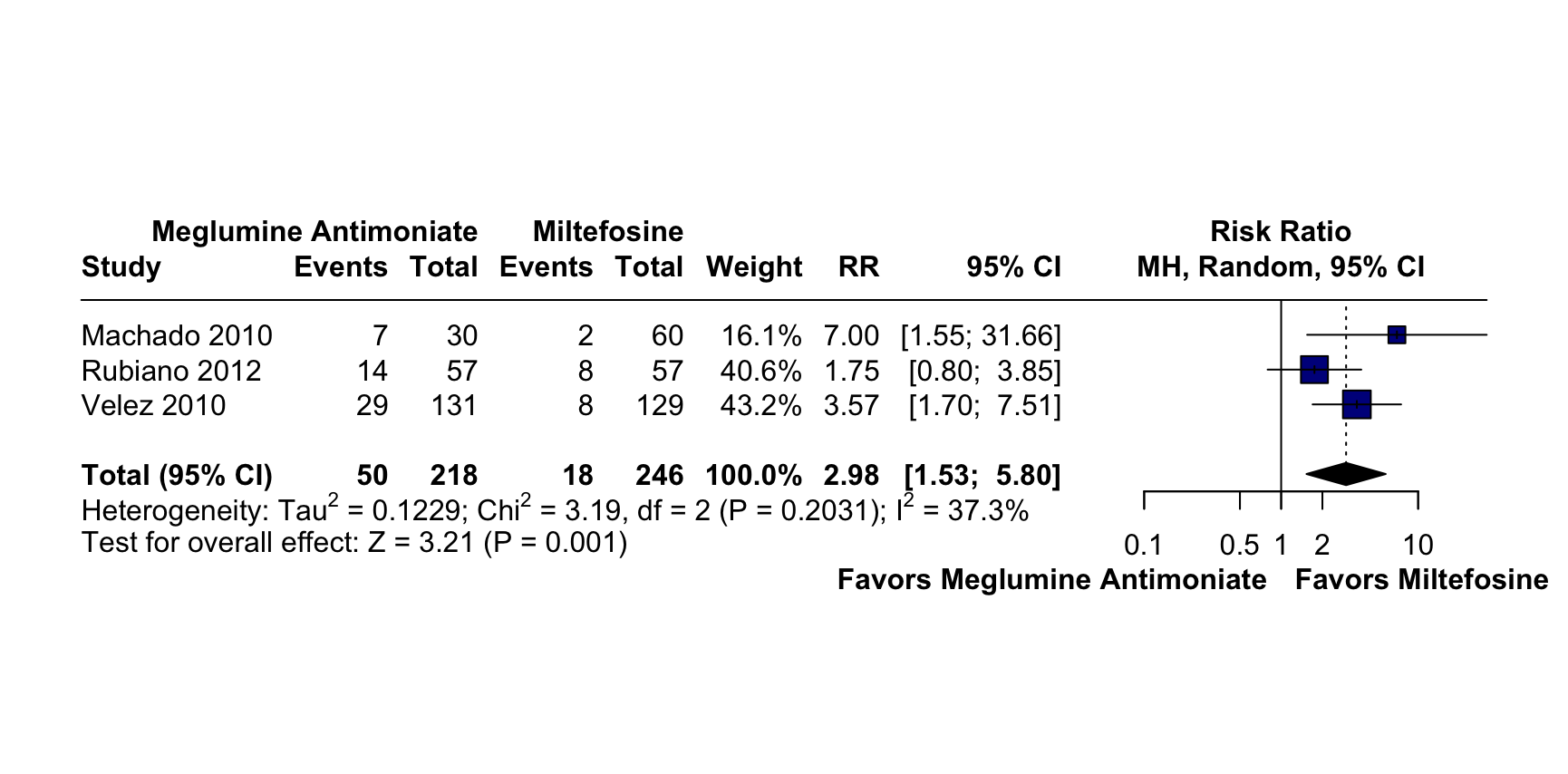


Three studies contributed data on fever occurrence.^2,5,8^ The pooled analysis showed that patients treated with meglumine antimoniate had a significantly higher risk of developing fever compared to those receiving miltefosine (RR 2.98; 95% CI 1.53–5.80; P = 0.001).

Supplemental Figure 21 Headache


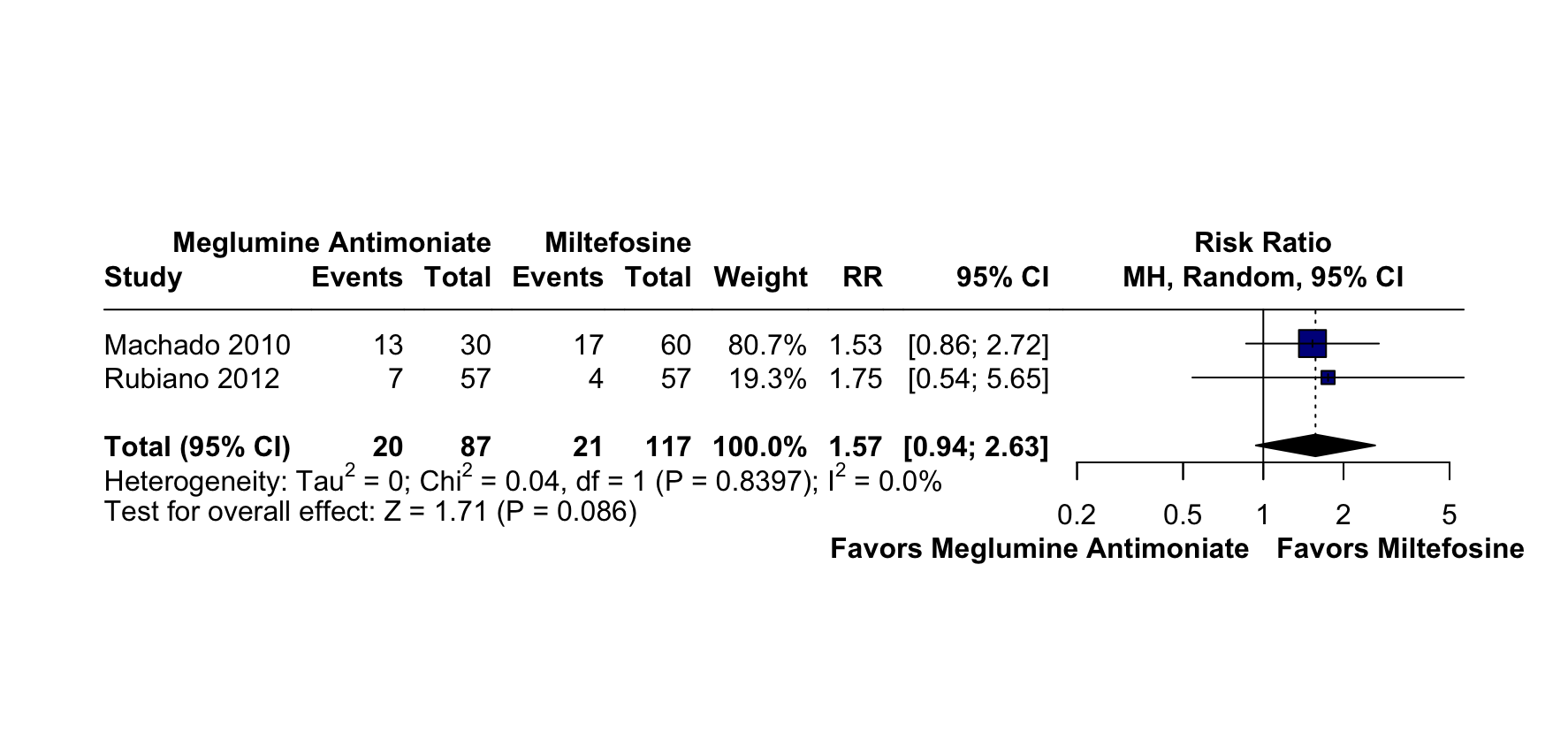


Two studies reported data on headache occurrence.^2,5^ The pooled risk ratio indicated no statistically significant difference between groups, although there was a trend toward a higher incidence of headache in the meglumine antimoniate group (RR 1.57; 95% CI 0.94–2.63; P = 0.086).

Supplemental Figure 22. Leave-one-out Vomiting


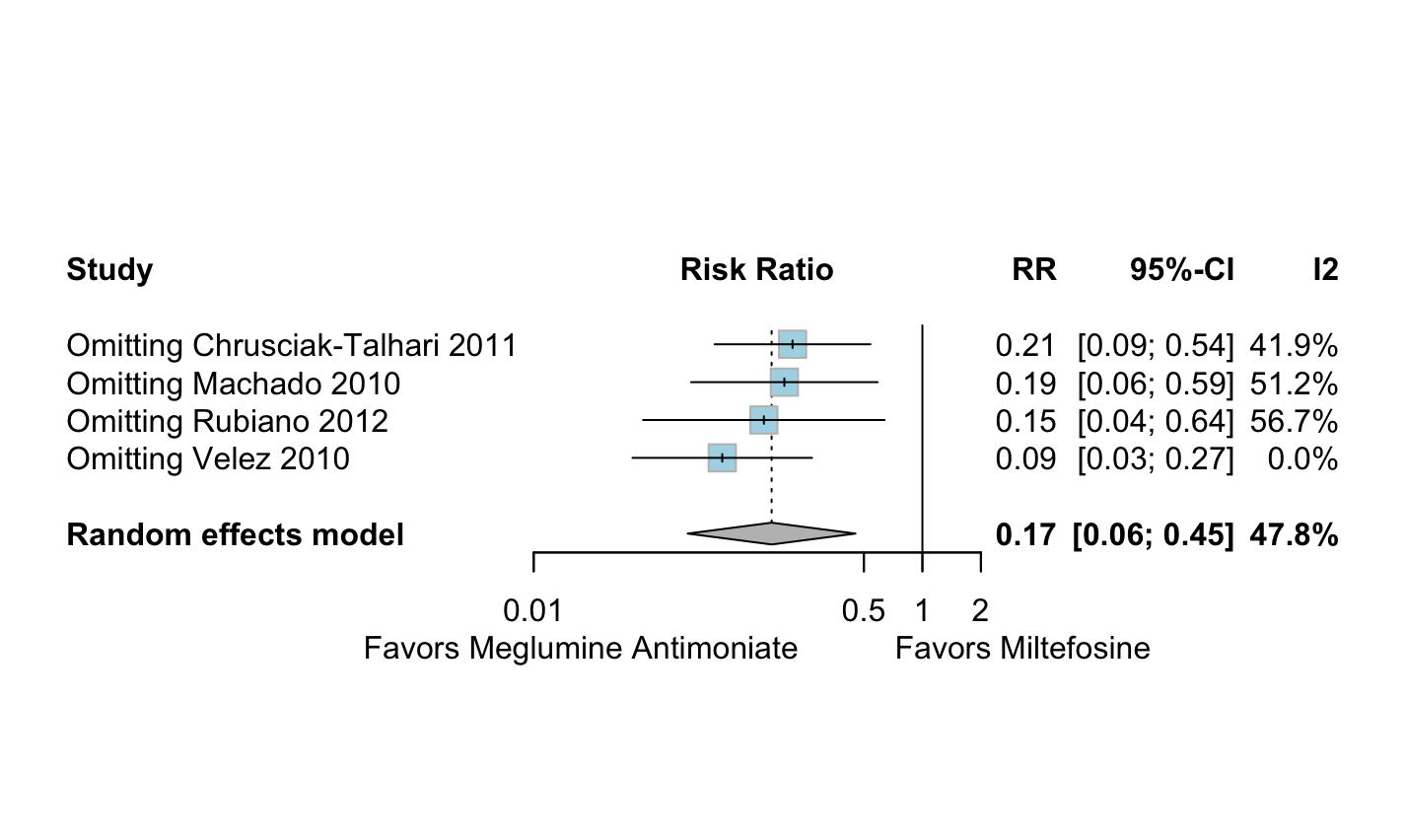


For vomiting, the leave-one-out sensitivity analysis showed that no single study significantly altered the overall effect estimate or heterogeneity. The pooled risk ratio (RR) remained robust, ranging from 0.09 to 0.21, with all confidence intervals consistently favoring meglumine antimoniate. The heterogeneity (I²) fluctuated between 0.0% and 56.7%, with the greatest reduction observed when omitting Velez et al. 2010, decreasing I² to 0%.^8^

Supplemental Figure 23. Baujat analysis Vomiting


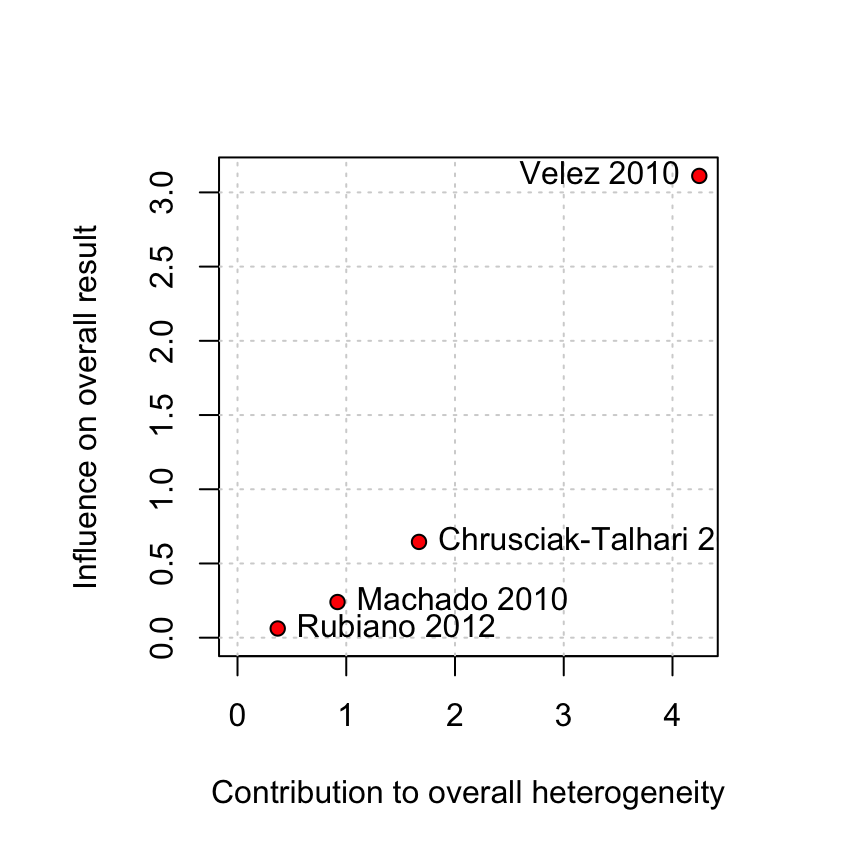


The Baujat plot identified Velez et al. 2010 as the primary contributor to both heterogeneity and influence on the overall result for vomiting.^8^ Although Chrusciak-Talhari et al. 2011 and Machado et al. 2010 also contributed modestly to heterogeneity, their influence on the overall effect estimate was minimal.^1,2^

Supplemental Figure 24. Leave-one-out Arthralgia


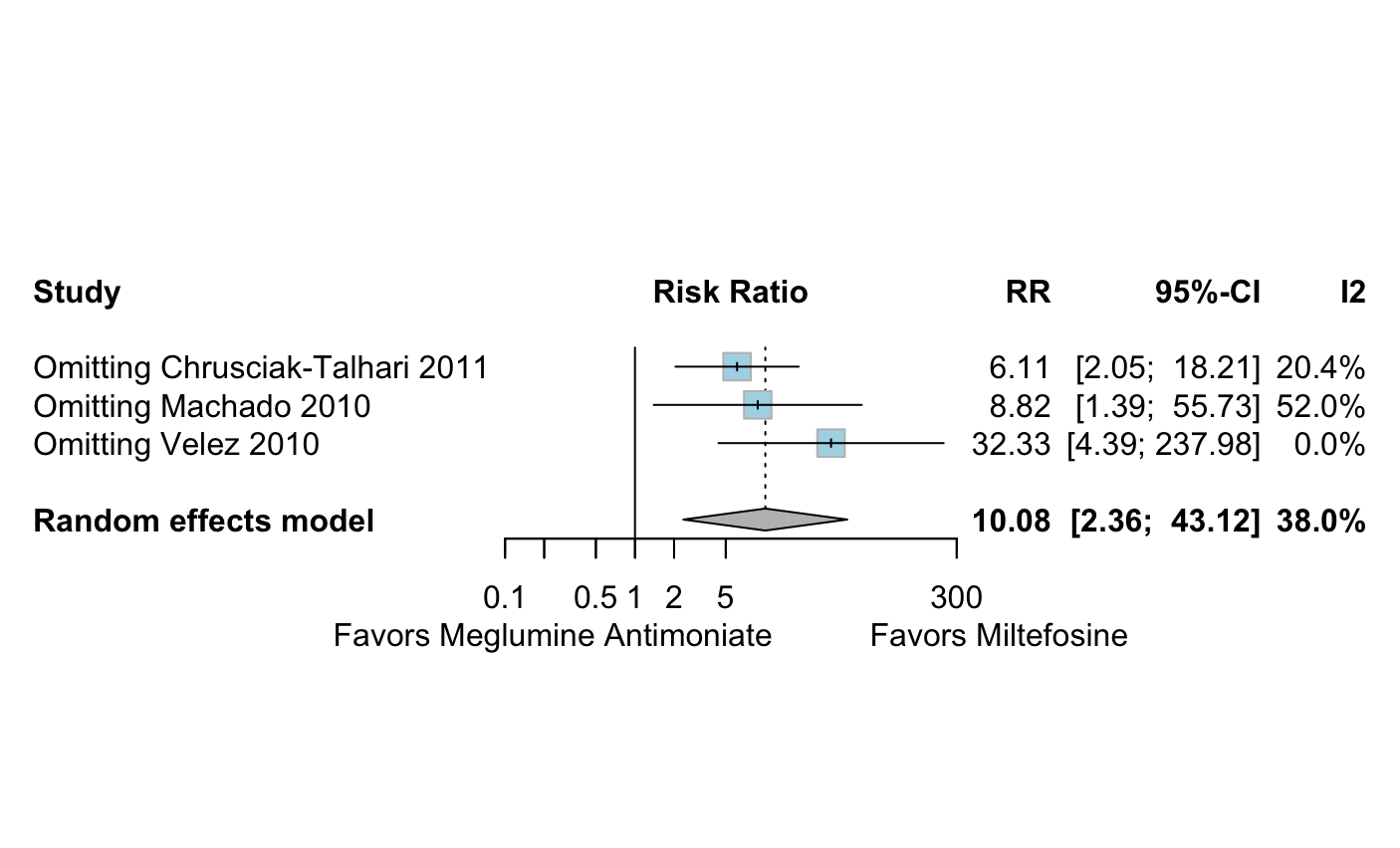


For arthralgia, the leave-one-out sensitivity analysis showed that no single study significantly altered the overall effect estimate or heterogeneity. The pooled risk ratio (RR) remained robust, ranging from 6.11 to 32.33, with all confidence intervals consistently favoring meglumine antimoniate. The heterogeneity (I²) fluctuated between 0.0% and 52.0%, with the greatest reduction observed when omitting Velez et al. 2010, decreasing I² to 0%.^8^

Supplemental Figure 25. Baujat Analysis Arthralgia


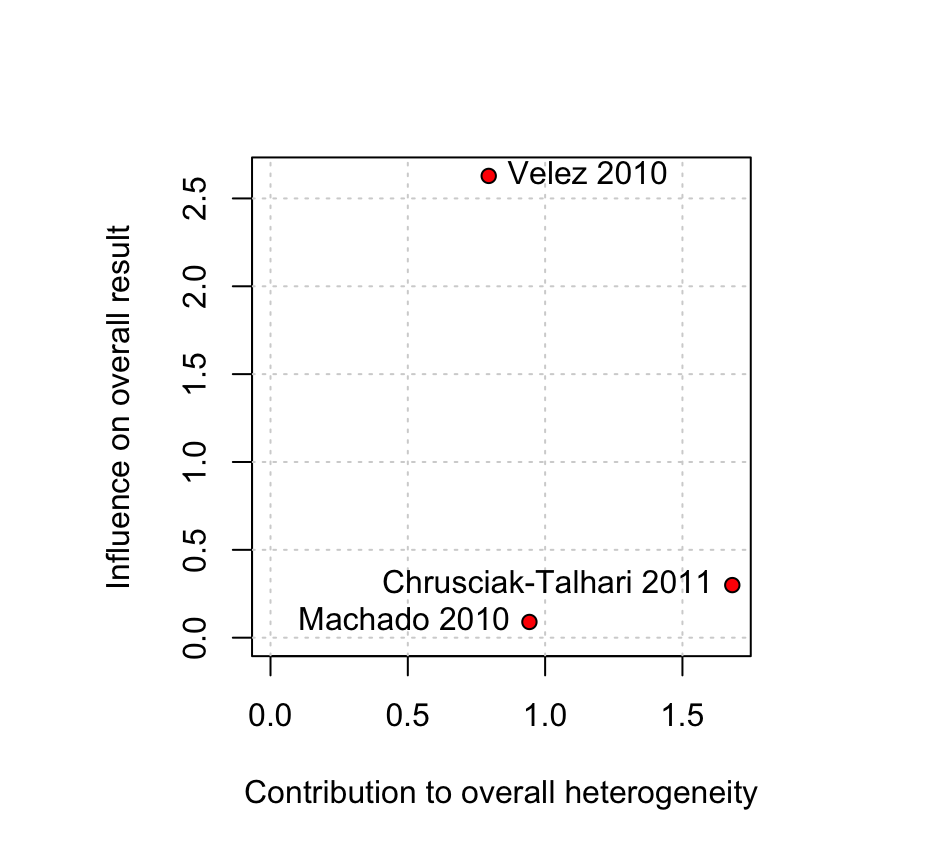


The Baujat plot indicated that Velez et al. 2010 contributed most to both heterogeneity and influence on the overall result. However, its influence did not substantially affect the robustness of the overall findings.^8^

Supplemental Figure 26. Leave-one-out Fever


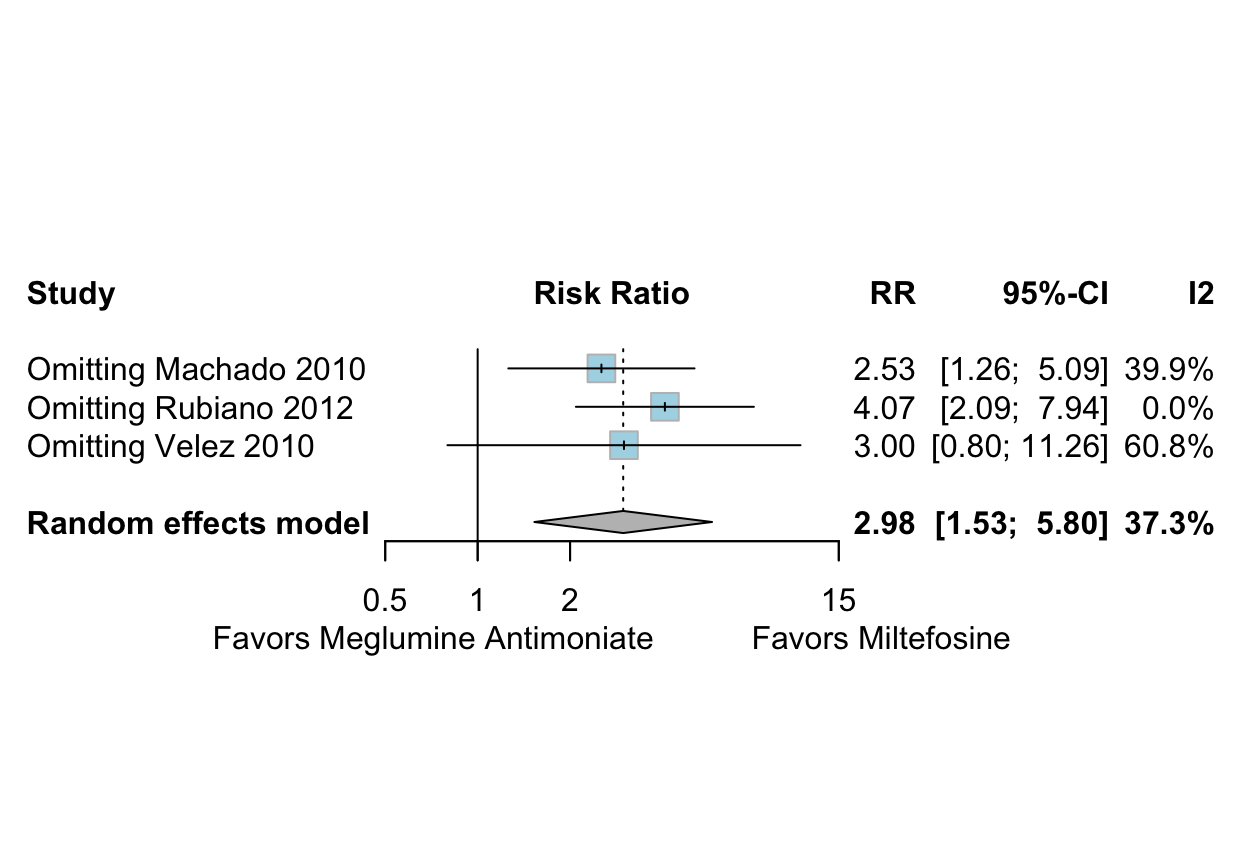


The leave-one-out sensitivity analysis demonstrated that no single study substantially altered the overall effect estimate or heterogeneity. The pooled risk ratio (RR) remained stable, ranging from 2.53 to 4.07, with all confidence intervals consistently favoring miltefosine. The heterogeneity (I²) varied from 0.0% to 60.8%, with the lowest heterogeneity observed when omitting Rubiano et al. 2012, reducing I² to 0%.^5^

Supplemental Figure 27. Baujat analysis Fever


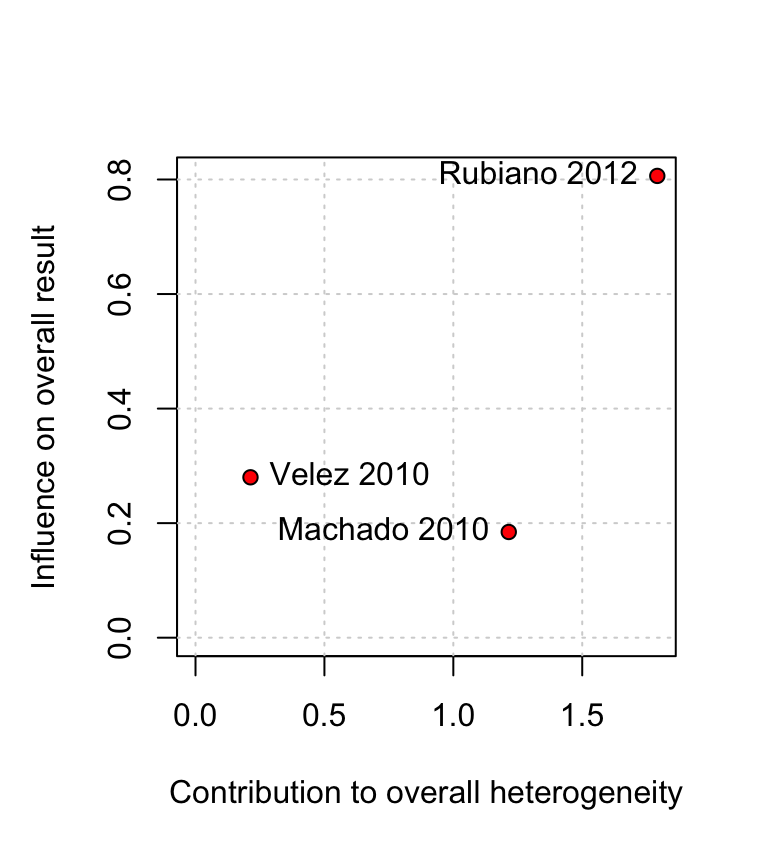


The Baujat plot showed that Rubiano 2012 contributed the most to the overall heterogeneity and had the greatest influence on the pooled effect size.^5^

**REFERENCES**

1. Chrusciak-Talhari A, Dietze R, Chrusciak Talhari C, da Silva RM, Gadelha Yamashita EP, de Oliveira Penna G, et al. Randomized controlled clinical trial to access efficacy and safety of miltefosine in the treatment of cutaneous leishmaniasis Caused by Leishmania (Viannia) guyanensis in Manaus, Brazil. Am J Trop Med Hyg. 2011 Feb;84:255-60. doi: 10.4269/ajtmh.2011.10-0155.
2. Machado PR, Ampuero J, Guimarães LH, Villasboas L, Rocha AT, Schriefer A, et al. Miltefosine in the treatment of cutaneous leishmaniasis caused by Leishmania braziliensis in Brazil: a randomized and controlled trial. PLoS Negl Trop Dis. 2010 Dec 21;4:e912. doi: 10.1371/journal.pntd.0000912.
3. Machado PRL, Prates FVO, Boaventura V, Lago T, Guimarães LH, Schriefer A, et al. A Double-blind, Randomized Trial to Evaluate Miltefosine and Topical Granulocyte Macrophage Colony-stimulating Factor in the Treatment of Cutaneous Leishmaniasis Caused by Leishmania braziliensis in Brazil. Clin Infect Dis. 2021 Oct 5;73:e2465-e2469. doi: 10.1093/cid/ciaa1337.
4. Mendes L, Guerra JO, Costa B, Silva ASD, Guerra MDGB, Ortiz J, et al. Association of miltefosine with granulocyte and macrophage colony-stimulating factor (GM-CSF) in the treatment of cutaneous leishmaniasis in the Amazon region: A randomized and controlled trial. Int J Infect Dis. 2021 Feb;103:358-363. doi: 10.1016/j.ijid.2020.11.183.
5. Rubiano LC, Miranda MC, Muvdi Arenas S, Montero LM, Rodríguez-Barraquer I, Garcerant D, et al. Noninferiority of miltefosine versus meglumine antimoniate for cutaneous leishmaniasis in children. J Infect Dis. 2012 Feb 15;205:684-92. doi: 10.1093/infdis/jir816.
6. Soto J, Rea J, Balderrama M, Toledo J, Soto P, Valda L, et al. Efficacy of miltefosine for Bolivian cutaneous leishmaniasis. Am J Trop Med Hyg. 2008 Feb;78:210-1.
7. Soto J, Toledo JT. Oral miltefosine to treat new world cutaneous leishmaniasis. Lancet Infect Dis. 2007 Jan;7:7. doi: 10.1016/S1473-3099(06)70665-X.
8. Vélez I, López L, Sánchez X, Mestra L, Rojas C, Rodríguez E. Efficacy of miltefosine for the treatment of American cutaneous leishmaniasis. Am J Trop Med Hyg. 2010 Aug;83:351-6. doi: 10.4269/ajtmh.2010.10-0060.
